# Determinants of response to CDK4/6 inhibitors in the real-world setting

**DOI:** 10.1038/s41698-023-00438-0

**Published:** 2023-09-13

**Authors:** Agnieszka K. Witkiewicz, Emily Schultz, Jianxin Wang, Deanna Hamilton, Ellis Levine, Tracey O’Connor, Erik S. Knudsen

**Affiliations:** 1 Department of Molecular and Cellular Biology, Roswell Park Comprehensive Cancer Center, Buffalo, NY 14203 USA; 2 Department of Pathology, Roswell Park Comprehensive Cancer Center, Buffalo, NY 14203 USA; 3 Department of Medicine, Roswell Park Comprehensive Cancer Center, Buffalo, NY 14203 USA

**Keywords:** Predictive markers, Breast cancer

## Abstract

Despite widespread use and a known mechanism of action for CDK4/6 inhibitors in combination with endocrine therapy, features of disease evolution and determinants of therapeutic response in the real-world setting remain unclear. Here, a cohort of patients treated with standard-of-care combination regimens was utilized to explore features of disease and determinants of progression-free survival (PFS) and overall survival (OS). In this cohort of 280 patients, >90% of patients were treated with palbociclib in combination with either an aromatase inhibitor (AI) or fulvestrant (FUL). Most of these patients had modified Scarff–Bloom–Richardson (SBR) scores, and ER, HER2, and PR immunohistochemistry. Both the SBR score and lack of PR expression were associated with shorter PFS in patients treated with AI combinations and remained significant in multivariate analyses (HR = 3.86, *p* = 0.008). Gene expression analyses indicated substantial changes in cell cycle and estrogen receptor signaling during the course of treatment. Furthermore, gene expression-based subtyping indicated that predominant subtypes changed with treatment and progression. The luminal B, HER2, and basal subtypes exhibited shorter PFS in CDK4/6 inhibitor combinations when assessed in the pretreatment biopsies; however, they were not associated with OS. Using unbiased approaches, cell cycle-associated gene sets were strongly associated with shorter PFS in pretreatment biopsies irrespective of endocrine therapy. Estrogen receptor signaling gene sets were associated with longer PFS particularly in the AI-treated cohort. Together, these data suggest that there are distinct pathological and biological features of HR+/HER2− breast cancer associated with response to CDK4/6 inhibitors. Clinical trial registration number: NCT04526587.

## Introduction

Hormone receptor-positive and HER2-negative (HR+/HER2−) breast cancer represents one of the most prevalent malignancies in the western world. The treatment of localized disease involves surgery, chemotherapy (if indicated), and radiation, which is followed by endocrine therapy in the adjuvant setting^1,2^. Gene expression signatures have been developed to determine the risk of recurrence for early-stage disease and to elucidate the benefit from chemotherapy or extended treatment with endocrine therapy^3–5^. These precision approaches are effective; however, a significant number of patients develop recurrent metastatic disease or present with metastatic disease de novo. HR+/HER2− recurrent metastatic breast cancer can develop over the course of many years and represents a continual risk^6,7^.

The treatment of metastatic HR+/HER2− breast cancer has evolved^1^. Historically, in post-menopausal women, endocrine therapy, either aromatase inhibitors (AI) or selective estrogen degraders (SERDS) were prescribed for the treatment of metastatic disease. These therapies can prolong progression-free survival (PFS), but responses are of limited duration and treatments are not curative. The PFS for the standard-of-care AI letrozole is ~14–16 months, while the PFS of the standard-of-care SERD fulvestrant is ~6–8 months^8^. Multiple clinical trials have interrogated the use of targeted agents in conjunction with endocrine therapy to enhance the durability of response^9^. These randomized trials supported the use of CDK4/6 inhibitors to limit disease progression and in certain settings yield an increase in overall survival (OS)^8,10–16^. Currently, three CDK4/6 inhibitors are FDA approved for treatment of metastatic HR+/HER2− breast cancer. These therapies generally double the PFS of endocrine therapy alone; however, there is a small group of patients (15–25%) that rapidly progress irrespective of the CDK4/6 inhibitor, suggesting the existence of tumors that are intrinsically resistant to CDK4/6 inhibitor and endocrine therapy combination treatment.

Preclinical studies have provided insights into determinants of response to CDK4/6 inhibition. It was shown that RB-deficient models of breast cancer are resistant to CDK4/6 inhibitors^17,18^. These models generally exhibit elevated expression of p16^Ink4a^ concomitant with the disruption of RB function^19^. While this state appears to be relatively common in triple negative breast cancer, loss of RB is infrequent in HR+/HER2− breast cancer^20–22^. However, direct analyses of RB-status indicated shorter PFS in patients with RB loss compared with wild-type counterparts^20,22^. Several different mechanisms have emerged related to resistance, including deregulation of Cyclin E, CDK6, RAS-pathway, AMBRA1, MYC expression, and HIPPO-pathway^21,23–27^. Notably, each of these genetic events ultimately compromise the activity of the CDK4/6 inhibitor in eliciting potent cell cycle arrest.

Markers used to predict response to CDK4/6 inhibitors have not yet been developed for clinical application^22,28^. In the context of adjuvant endocrine therapy, several gene expression panels are commonly used.^3,29,30^. These signatures generally define the risk of recurrence, the corresponding benefit from chemotherapy, and/or the need for longer treatment with endocrine therapy. Interestingly, most of these signatures harbor proliferation-associated genes that are regulated by the RB pathway^31,32^. For example, Oncotype Dx is comprised of modules that largely interrogate estrogen receptor signaling and proliferation status; similarly, PAM50 intrinsic subtyping utilizes proliferation-associated genes to differentiate luminal A and B^5,32^. Therefore, several different signatures that sample the RB pathway aberrations are prognostic in cohorts of HR+/HER2− breast cancer^33–37^. Importantly, due to the routine sampling of breast cancer to evaluate markers (i.e., estrogen receptor and HER2), most biomarker strategies remain based on tissue specimens.

Due to the CDK4/6 inhibitor mechanism of action, there are several suspected determinants of response, which would include RB loss and p16^Ink4a^ over-expression. While RB loss does appear to be associated with short PFS with CDK4/6 inhibitors^22^, it is a rare event in metastatic CDK4/6 inhibitor-naive breast cancer. Similarly, Cyclin E gene expression has been associated with shorter PFS^23^. A variety of biomarker analyses from randomized clinical trials have identified putative determinants for the duration of PFS^26,38–40^. However, analyses of features of response in the real-world standard-of-care setting, incorporating standard pathological and histological markers have yet to be evaluated. Here, we examined a cohort of patients treated with CDK4/6 inhibitors to delineate features of evolution during treatment as potential determinants of PFS that could be applicable to standard practice.

## Results

### Patient cohort and analysis of standard histological and pathological markers

**Fig. 1:**
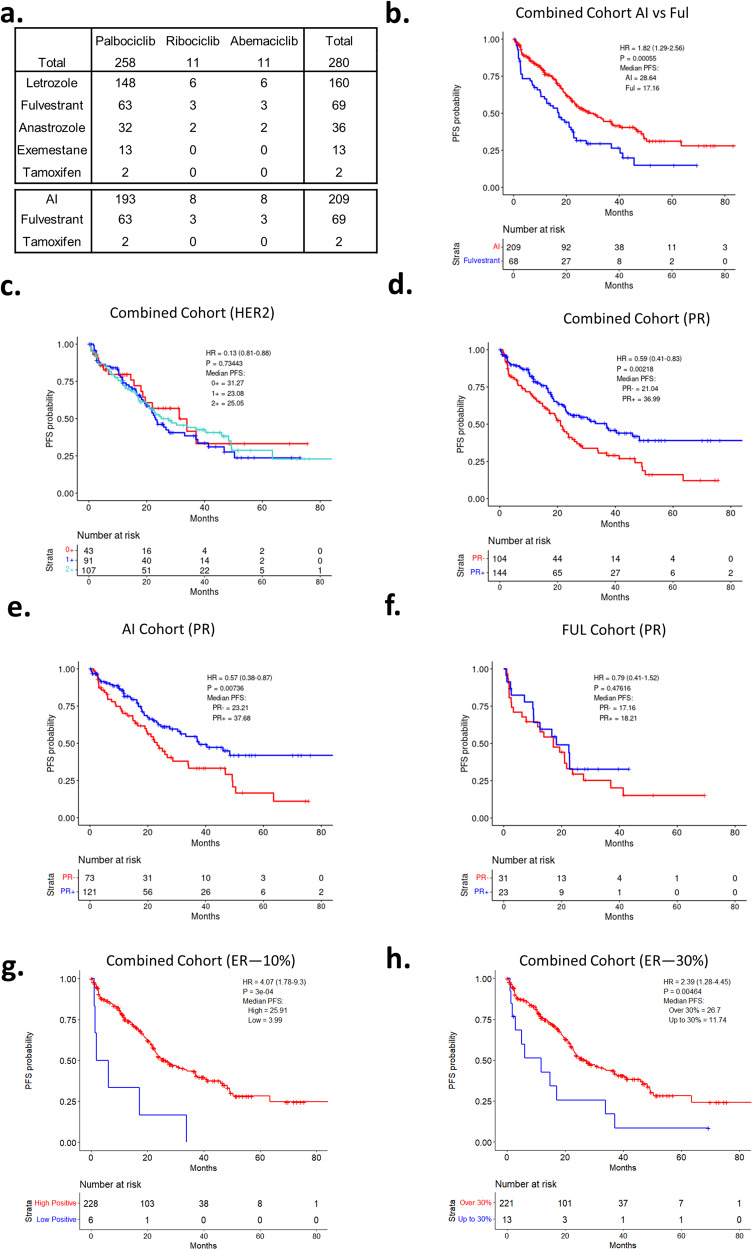
Patient cohort and analysis of pathologic markers. **a** Table summarizing combination of CDK therapy and hormonal therapy per patient. **b** Kaplan–Meier analysis of progression-free survival comparing CDK4/6i combinations with fulvestrant vs AI. *p* = 0.00055 by log rank. **c** Kaplan–Meier analysis of progression-free survival comparing HER2 subtype resulted by IHC across all patients. *p* = 0.73 by log rank. **d** Kaplan–Meier analysis of progression-free survival comparing PR status across all patients. *p* = 0.0022 by log rank. **e** Kaplan–Meier analysis of progression-free survival comparing PR status for patients taking AI. *p* = 0.0074 by log rank. **f** Kaplan–Meier analysis of progression-free survival comparing PR status for patients taking fulvestrant. *p* = 0.48 by log rank. **g** Kaplan–Meier analysis of progression-free survival comparing ER status split at 10% staining intensity across all patients. *p* = 3e^−4^ by log rank. **h** Kaplan–Meier analysis of progression-free survival comparing ER status split at 30% staining intensity across all patients. *p* = 0.005 by log rank.

In order to define determinants of the response to CDK4/6 inhibitors used in the standard-of-care setting, an IRB approved study was developed (NCT04526587) in parallel with retrospective chart-review. Over 3500 patients were screened as summarized in the CONSORT diagram (Supplementary Fig. 1). A total of 280 patients were evaluated as of December 2022 (Table 1). Detailed clinical and pathological information was obtained by chart review and abstracted to a REDCap database. In this patient population, the majority (92%) were treated with palbociclib along with either an aromatase inhibitor (AI) or fulvestrant (FUL) as summarized (Fig. 1a). In this cohort, the progression-free survival (PFS) for patients treated with the AI or FUL combinations were 28.6 and 17.2 months, respectively (Fig. 1b), which is comparable to that observed in randomized clinical trials^8,41,42^. Among clinical variables, visceral involvement, prior endocrine therapy and recurrent disease were associated with shorter PFS (Supplementary Fig. 1), consistent with other studies. In this cohort, the OS was determined from either the initiation of treatment or the point of progression. From the initiation of treatment, the AI-treated group had a longer OS (Supplementary Fig. 1); however, when using the point of progression on CDK4/6 inhibitor based therapy as the starting point, the OS for each treatment group was veritably identical (median OS ~19 months) (Supplementary Fig. 1). As may be expected, in this cohort the post-progression therapy was highly varied (Supplementary Fig. 1).

**Table 1:** Patient characteristics.

Demographic	All patients	AI (%)	Fulvestrant (%)
	*n* = 280	*n* = 209 (74.64)	*n* = 69 (24.64)
Age at CDK start, years			
<50	51	42	9
≥50	228	167	59
ECOG at CDK start			
0	144	110	34
1	107	76	29
2	16	12	4
3	2	2	0
Sex			
Female	275	204	69
Male	5	5	0
Race/ethnicity			
European	242	185	55
Asian	3	2	1
African American	25	15	10
Hispanic & Latino	3	1	2
Other	2	2	0
Menopause status at CDK start			
Pre & Peri	49	43	6
Post	222	160	60
Male	5	5	0
Metastatic status			
Visceral	133	92	40
Non-visceral	147	117	29
Metastatic status at presentation			
De novo	91	81	9
Recurrent	189	128	60
Number of metastatic sites			
1	124	92	32
2	86	61	23
≥3	56	45	11
Prior endocrine therapy			
Yes	183	118	63
No	97	91	6
Prior chemotherapy			
Yes	144	98	46
No	136	111	23

Table showing various demographic characteristics for the patients included in the study.

*AI* aromatase inhibitor.

To determine if any standard pathological marker was associated with PFS, we assessed progesterone receptor (PR) and HER2 expressions. We found that the level of HER2 as determined by IHC scoring (0, 1+, or 2+/FISH non-amplified) was not associated with PFS in the combined cohort (Fig. 1c) or in sub-group analysis of AI or FUL treated patients (Supplementary Fig. 2). In contrast, PR status was associated with PFS, with PR negative/low status denoting significantly shorter PFS in the combined cohort (Fig. 1d). In subgroup analyses the absence of PR was specifically associated with PFS in the AI-treated patients, but not in the FUL-treated patients (Fig. 1e, f). Since PR expression is a surrogate for ER activity, we also assessed the influence of ER status on PFS. While all of the cases are ER positive, a small subset have low positivity (<10%). This state was associated with shorter PFS (Fig. 1g), similarly low/mid levels of ER staining (<30%) were associated with shortened PFS (Fig. 1h).

**Fig. 2:**
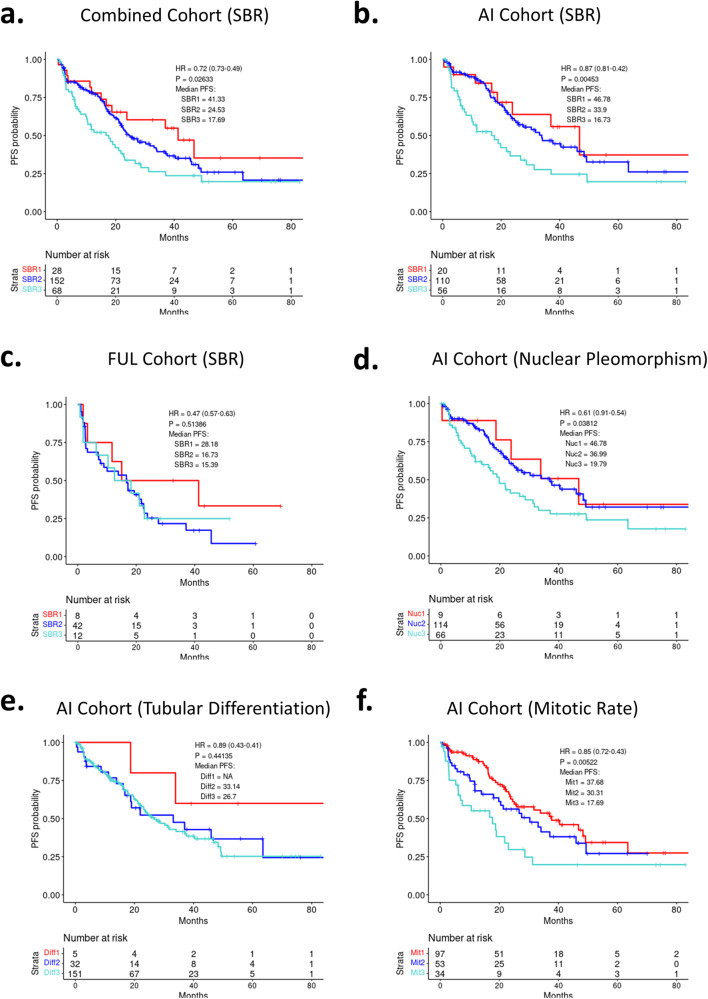
Analysis of SBR score components. **a** Kaplan–Meier analysis of progression-free survival comparing overall SBR score across all patients. *p* = 0.026 by log rank. **b** Kaplan–Meier analysis of progression-free survival comparing overall SBR score across patients taking AI. *p* = 0.0045 by log rank. **c** Kaplan–Meier analysis of progression-free survival comparing overall SBR score across patients taking fulvestrant. *p* = 0.51 by log rank. **d** Kaplan–Meier analysis of progression-free survival comparing Nuclear Pleomorphism across patients taking AI. *p* = 0.038 by log rank. **e** Kaplan–Meier analysis of progression-free survival comparing Tubular Differentiation across patients taking AI. *p* = 0.44 by log rank. **f** Kaplan–Meier analysis of progression-free survival comparing Mitotic Rate across patients taking AI. *p* = 0.0052 by log rank.

Most patients (*n* = 248) had tumor histologic grade assigned using the Nottingham modification of Scarff–Bloom–Richardson (SBR) scoring. Higher SBR score was associated with shorter PFS in the context of the combined cohort (Fig. 2a) as well as in the AI treated subgroup (Fig. 2b), but not in the FUL treated group (Fig. 2c). Since different tissue specimens (resection and biopsies) were utilized for the determination of the SBR, tissue obtaining procedures were also evaluated and did not significantly alter the association with PFS (not shown). SBR is composed of tubular differentiation, nuclear pleomorphism and mitotic rate grades; each individual component was analyzed in the combined cohort (Supplementary Fig. 3) as well as in the AI treated group (Fig. 2d–f). In this context, the mitotic rate and nuclear pleomorphism grades were associated with outcome, but not the tubular differentiation. The SBR score variables were not associated with PFS in the FUL sub-group (Supplementary Fig. 3). Univariate analyses of the pathological and histological markers are summarized in Table 2. Employing a multivariate model that incorporated the significant markers, in concert with visceral metastatic disease and prior endocrine therapy, PR and SBR maintained significance (Table 2).

**Table 2:** Analysis of pathological and histological markers.

Univariate in the combined, AI, and FUL cohorts												
Comparison (v1 vs v2)	N1	N2	HR	*p* value	N1	N2	HR	*p* value	N1	N2	HR	*p* value
HER2 0+ vs HER2 1+	43	91	1.2392	0.445	39	66	0.889	0.704	4	25	3.860	0.188
HER2 0+ vs HER2 2+	43	107	1.1357	0.645	39	82	0.8857	0.682	4	25	3.532	0.221
PR− vs PR+	104	144	0.5859	0.00246	73	121	0.5736	0.00814	31	23	0.7865	0.477
Non-visceral vs visceral metastasis	146	132	1.4196	0.0331	117	92	1.3240	0.164	29	40	1.3728	0.281
Distant Mets vs local disease	234	14	0.4116	0.0801	198	11	0.4751	0.204	66	3	0.3024	0.239
No prior endocrine vs prior endocrine therapy	97	181	1.9834	0.00041	91	118	1.7144	0.0123	6	63	2.2928	0.165
SBR1 vs SBR2	28	152	1.3715	0.2907	20	110	1.2392	0.5736	8	42	1.7402	0.254
SBR1 vs SBR3	28	69	2.0574	0.0227	20	56	2.3817	0.0272	8	13	1.5936	0.407
Tubular differentiation 1 vs 2	10	40	1.1825	0.716	5	32	2.3227	0.260	5	8	0.7368	0.649
Tubular differentiation 1 vs 3	10	202	1.4305	0.394	5	151	2.4321	0.216	5	51	1.2789	0.642
Nuclear pleomorphism 1 vs 2	15	149	1.203	0.602	9	114	1.0953	0.847	6	35	1.9587	0.218
Nuclear pleomorphism 1 vs 3	15	89	1.704	0.138	9	66	1.8368	0.200	6	23	1.7881	0.302
Mitotic rate 1 vs 2	134	69	1.2001	0.353	97	53	1.3929	0.17457	37	16	0.9408	0.857
Mitotic rate 1 vs 3	134	42	1.5627	0.0535	97	34	2.3281	0.00154	37	8	0.5587	0.277

Comparison (v1 vs v2)	N1	N2	HR	Covariate *p* value	Model *p* value
Multivariate in the combined cohort					
PR− vs PR+	104	144	0.6019	0.0062	4e^−5^
Non-visceral vs visceral metastasis	146	132	1.2441	0.24502	
No prior endocrine vs prior endocrine therapy	97	181	1.6544	0.0209	
SBR1 vs SBR2	28	152	1.6633	0.13532	
SBR1 vs SBR3	28	69	2.7842	0.00404	
Multivariate in the AI cohort					
PR− vs PR+	73	121	0.5739	0.0116	3e^−4^
Non-visceral vs visceral metastasis	117	92	1.2368	0.3490	
No prior endocrine vs prior endocrine therapy	91	118	1.3940	0.1602	
SBR1 vs SBR2	20	110	1.3018	0.5179	
SBR1 vs SBR3	20	56	2.6599	0.0202	
Multivariate in the FUL cohort					
PR− vs PR+	31	23	0.8479	0.6587	0.4
Non-visceral vs visceral metastasis	29	40	0.8806	0.7306	
No prior endocrine vs prior endocrine therapy	6	63	2.9086	0.1751	
SBR1 vs SBR2	8	42	3.1049	0.0769	
SBR1 vs SBR3	8	13	3.3841	0.0848	

Univariate and multivariate analysis of common markers measured in ER+/HER2− breast cancer within the combined, AI, and fulvestrant cohorts of patients.

**Fig. 3:**
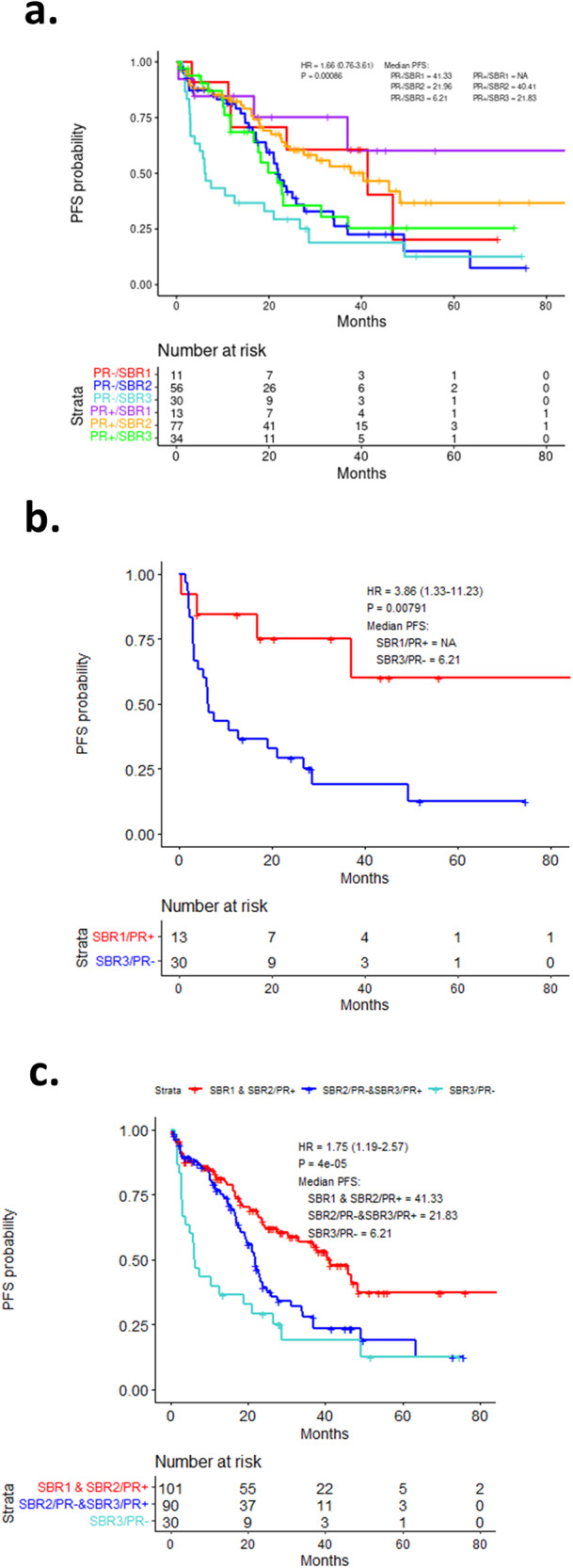
Analysis of SBR and PR subgroups. **a** Kaplan–Meier analysis of progression-free survival comparing PR status and overall SBR score across all patients. *p* = 0.00086 by log rank. **b** Kaplan–Meier analysis of progression-free survival comparing PR/SBR extremes across all patients. *p* = 0.0079 by log rank. **c** Kaplan–Meier analysis of progression-free survival comparing various PR/SBR subgroups across all patients. *p* < 0.0001 by log rank.

These findings suggest that PR and SBR status could be utilized in combination to define tumors with a predicted long vs short PFS. In the combined cohort, combining PR and SBR status was significantly associated with PFS (Fig. 3a). In particular, the combination of SBR3 and PR-negative status was associated with an exceedingly short PFS, while being SBR1 and PR-positive was associated with a longer duration of PFS (Fig. 3b). Other combinations of SBR and PR were associated with intermediate PFS (Fig. 3c). As expected the SBR and PR combined scores were particularly relevant in the AI + CDK4/6 inhibitor-treated cohort (Supplementary Fig. 4).

### Tumor evolution

**Fig. 4:**
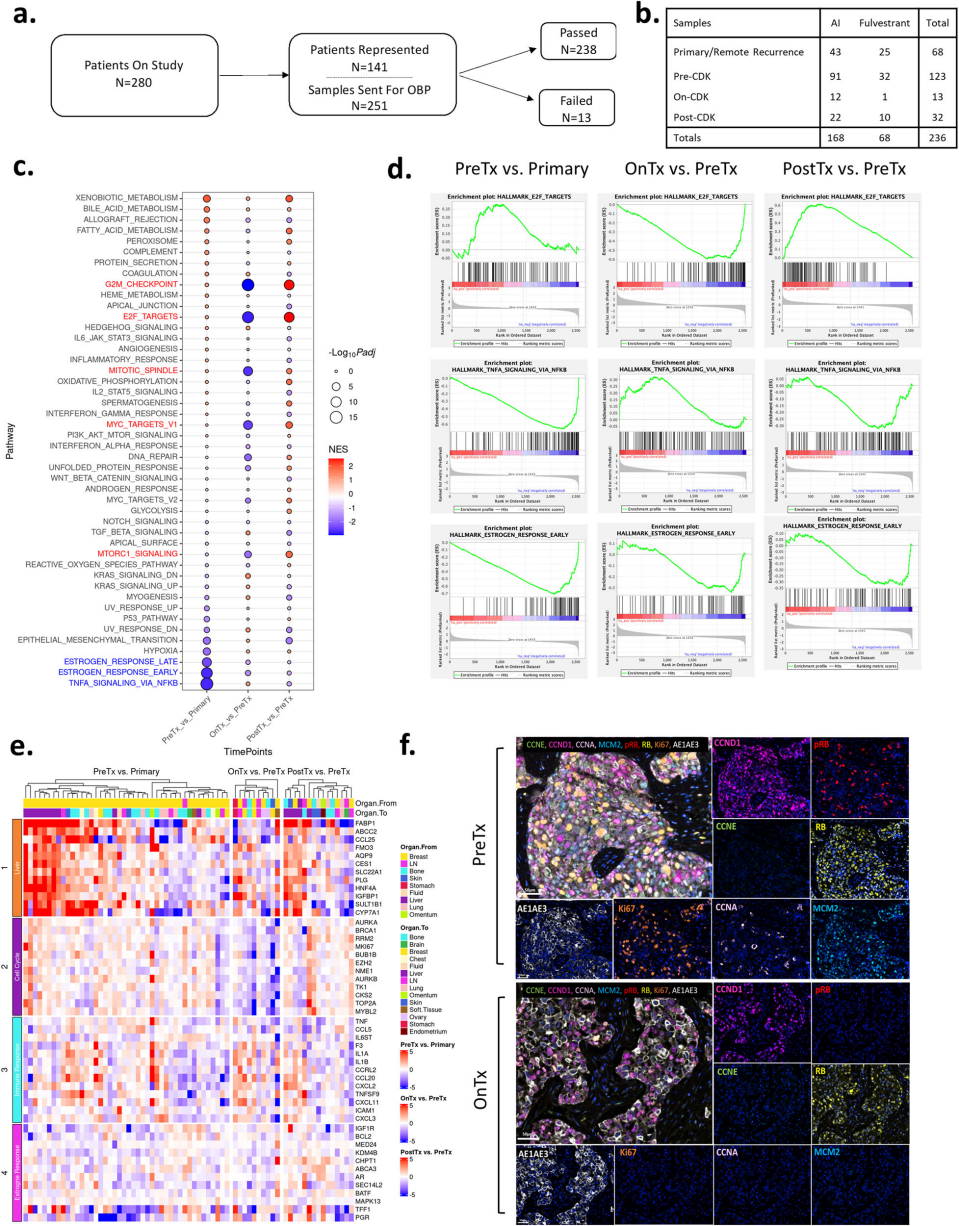
Tumor Evolution of Sequenced Samples. **a** Flowchart representing study samples sent for OBP. **b** Table summarizing tissue timepoint for all AI and fulvestrant samples passing OBP. **c** Enriched gene sets for differentially expressed genes between different timepoints using paired samples from the same patients. Log2 fold-change are calculated using DESeq2 in the paired mode setting and used as ranked gene list for input to GSEA. The normalized enrichment score (NES) along with associated *p* values are used to generate this bubble plot. **d** GSEA enrichment plots for select gene sets under each of the three timepoint comparisons. **e** Heatmap depicting gene expression fluctuations for select genes under different functional groups during the course of therapy. **f** Multispectral immunofluorescence imaging of a paired pretreatment and on-treatment sample.

Tumor tissue was obtained in the standard-of-care clinical treatment from 141 patients (e.g., from clinically mandated biopsies). Since multiple tissues may be available for a given clinical case, a total of 251 samples were employed for targeted gene expression analyses using the HTG Oncology Biomarker Panel of 2549 genes, of which 238 samples passed quality control (Fig. 4a and Supplementary Fig. 1). These tissues were subdivided based on treatment (2 tissues from tamoxifen-treated tumors were excluded), as well as the clinical stage/timepoint at which the tissue was obtained (Fig. 4b). Primary and remote recurrence samples represent tissue that were obtained significantly prior to receiving a CDK4/6 inhibitor-based therapy (mean = 78.2 months), pretreatment tissues were prior to the start of treatment (mean = 3.6 months), on treatment cases were while the patient was receiving therapy, whereas post-progression was after clinical progression on the CDK4/6 inhibitor-based therapy (Fig. 4b). To determine features associated with disease through treatment, paired samples from the same patient were utilized to define differentially expressed genes between primary/remote recurrence and pretreatment biopsies, as well as on-treatment and post-treatment. Ranked gene set enrichment analysis (GSEA) was deployed to investigate biological features associated with each step of the disease landscape (Fig. 4c). Notably, with progression to metastatic disease there are decreases in estrogen response signaling and TNF signaling via NF Kappa B (NFKB) (denoted in blue). There is an increase in terms related to xenobiotic and bile acid metabolism and weak induction of cell cycle related signatures (denoted in red) (Fig. 4c, d). On treatment there is profound inhibition of cell cycle signatures and modest induction of TNF signaling genes (Fig. 4c, d). Lastly, with progression there is further deregulation of cell cycle and decrease of estrogen receptor signaling and TNF signaling gene sets (Fig. 4c, d). To explore gene/signature level difference, heatmaps were used to evaluate changes in gene expression with regard to metastatic site and other features of disease (Fig. 4e). These analyses indicated that the xenobiotic metabolism and similar gene sets are associated with liver metastasis, indicating the importance of having detailed clinical information to define the basis of selective signatures within the data. Changes in cell cycle, TNF signaling, and estrogen signaling were relatively general features and not restricted to a particular metastatic site. To evaluate on-treatment and post-progression changes, they were compared against data from NeoPalAna where patients were treated with neo-adjuvant palbociclib in combination with anastrozole^43^. These data illustrated similar fluxes in gene expression on treatment which are reversed following progression (Supplementary Fig. 5). Lastly, to assess on-treatment effects related to cell cycle proteins, we utilized multispectral immunofluorescence staining on a subset of paired samples (Fig. 4f and Supplementary Fig. 5). These data showed the selective loss of phosphorylated RB with treatment, while total protein was retained consistent with the action of CDK4/6 inhibitors^44^. The downstream targets MCM2, Cyclin A, and Ki67 were suppressed, in agreement with the effect on expression of these genes on treatment. Cyclin D1 which is known to be retained in context of CDK4/6 inhibitor treatment^44^, was present in both pre- and on-treatment specimens.

### Intrinsic subtypes and association with PFS and OS

**Fig. 5:**
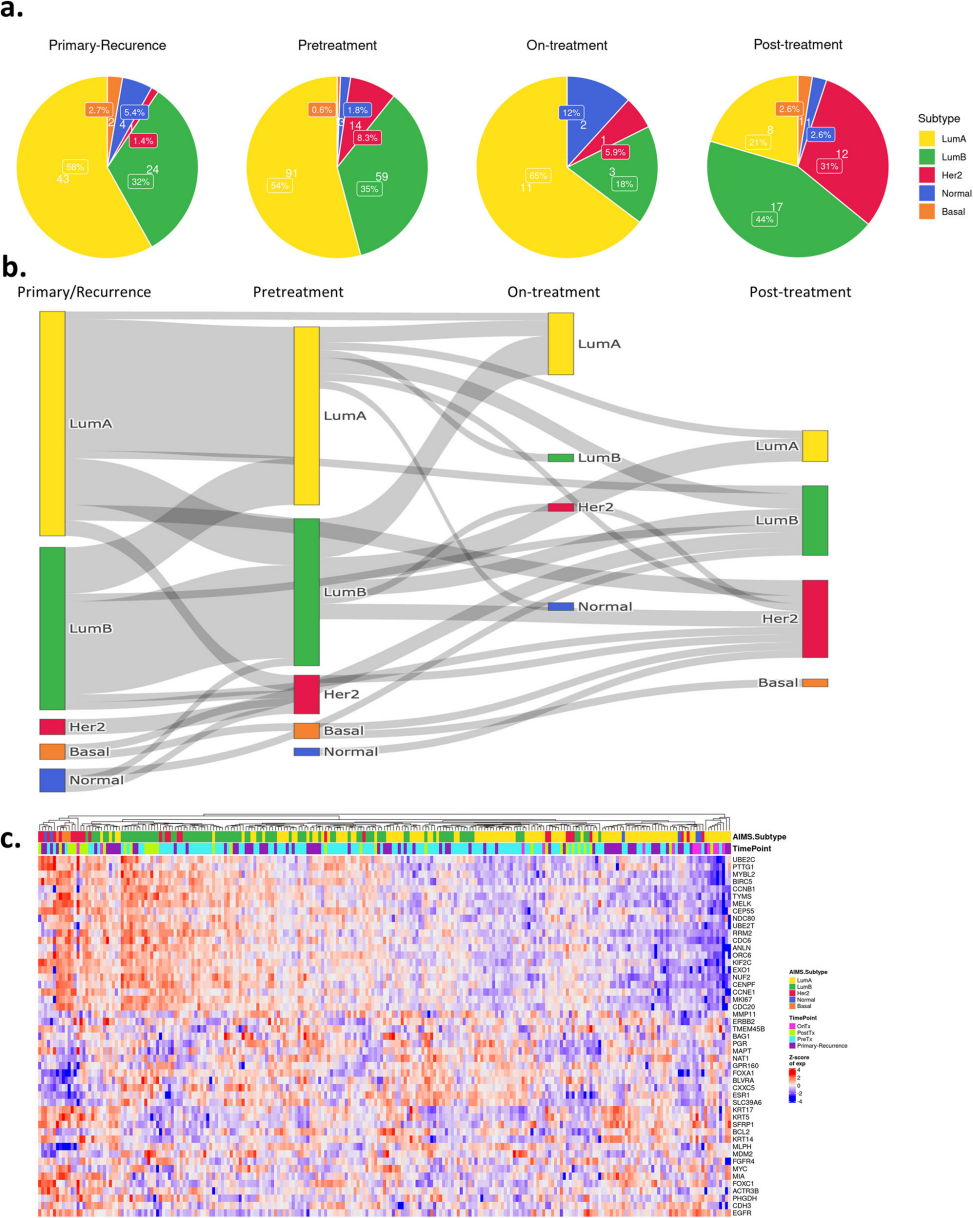
AIMS subtype evolution of sequenced samples. **a** Pie charts comparing breast cancer subtypes across tissue timepoints as predicted by Absolute Assignment of Breast Cancer Intrinsic Molecular Subtype (AIMS). **b** Sankey plot comparing breast cancer subtypes across tissue timepoints as predicted by AIMS. **c** Heatmap showing activation/suppression of genes in the PAM50 gene set. The AIMS predicted subtypes showed strong association with the gene expression programs of patients using this biomarker.

The Absolute Assignment of Breast Cancer Intrinsic Subtypes (AIMS)^45^ package was utilized to assign the subtype of each specimen. As expected, most tumors were scored as luminal A or B. The primary tumor/remote recurrences prior to CDK4/6 therapy start, exhibited a similar frequency of luminal A and B subtypes as observed in the pretreatment metastatic setting (Fig. 5a). While the number of on-treatment biopsies was limited, they were dominated by luminal A/normal subtype (Fig. 5a). Lastly, in post-progression disease, luminal A was observed in the minority of tumors (Fig. 5a). These data suggest that during treatment there are fluctuations of the subtypes, which is illustrated in the Sankey analysis showing the trajectories between paired samples (Fig. 5b). To further evaluate changes at the gene level, the PAM50 signature was assessed within all samples (Fig. 5c). These analyses show the general association of post-progression samples with high expression of cell cycle genes (e.g., PTTG1, UBE2C, and CCNB1) and lower expression of genes associated with estrogen signaling (e.g., PGR, ESR1, and FOXA1).

**Fig. 6:**
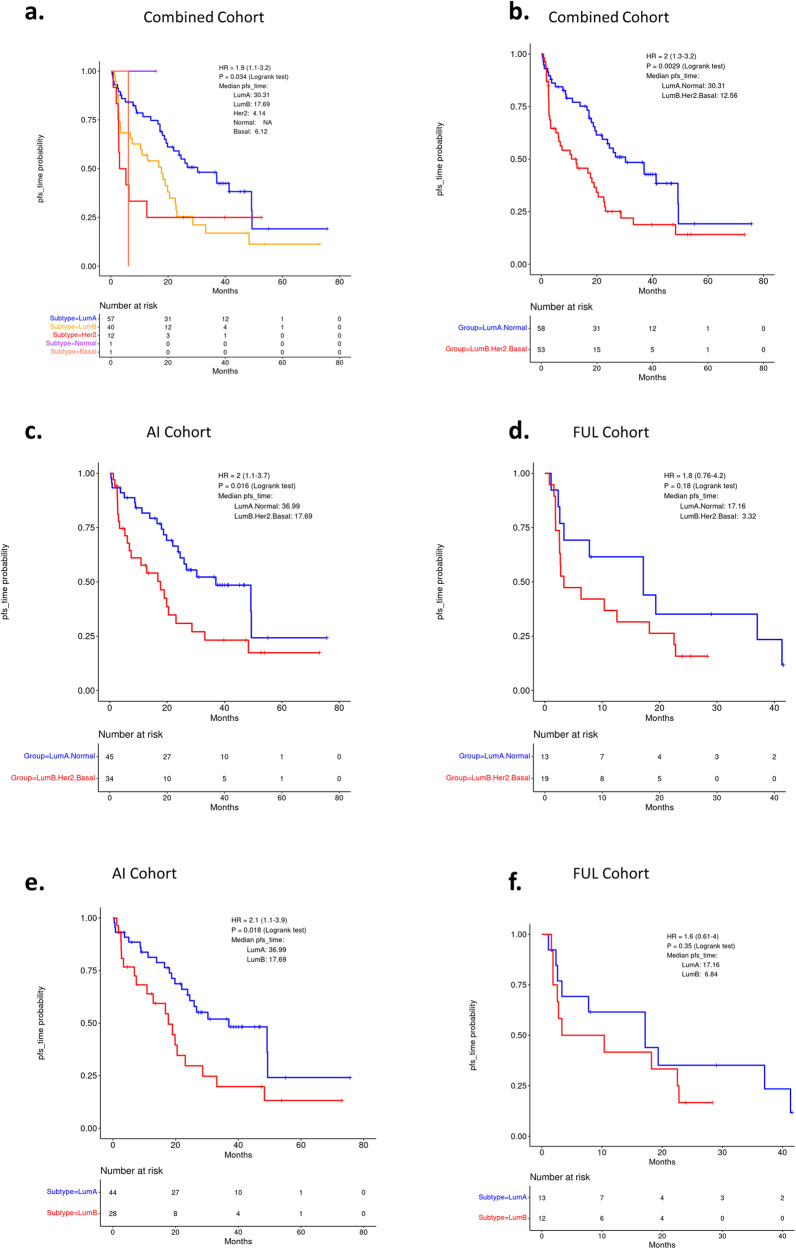
PFS of subtype subgroups. Testing for association of AIMS predicted Intrinsic subtypes with PFS. **a** Combined (AI and fulvestrant treated) cohorts for individual subtypes. *p* = 0.034 by log rank test. **b** Combined cohort on luminal A and normal, luminal B, Her2 and basal combined groups. *p* = 0.0029 by log rank test. **c**, **d** AI- or fulvestrant-treated cohorts separately on combined subtypes. *p* = 0.016 and *p* = 0.18, respectively, by log rank test. **e**, **f** AI- or fulvestrant-treated cohorts separately between luminal A and luminal B subtypes. *p* = 0.018 and *p* = 0.35, respectively, by log rank test.

In the analysis of the relationship of the subtypes with PFS, pretreatment samples were initially employed. In this setting, the AIMS subtypes were associated with PFS (Fig. 6a). Separating historically poor prognostic subtypes (luminal B, basal, and HER2) from better prognostic subtypes (luminal A and normal) illustrated association with PFS in the full cohort (Fig. 6b). This stratification was significant in subgroups of patients treated with AI, but not FUL although this could be due to the relatively small number of cases in the FUL sub-group (Fig. 6c, d). Similarly, the stratification of luminal A vs luminal B was significant only in the AI treated group (Fig. 6e, f). When applied to the remote recurrence/primary tumor samples, the AIMS subtypes only trended toward significance supporting the potential importance of evaluating the pretreatment metastatic samples (Supplementary Fig. 6). Interestingly, in evaluating the duration of OS, subtypes were only modestly associated with survival, albeit the non-luminal subtypes invariably associated with poorer outcome (Supplementary Fig. 6).

### CDK regulatory genes and duration of PFS

**Fig. 7:**
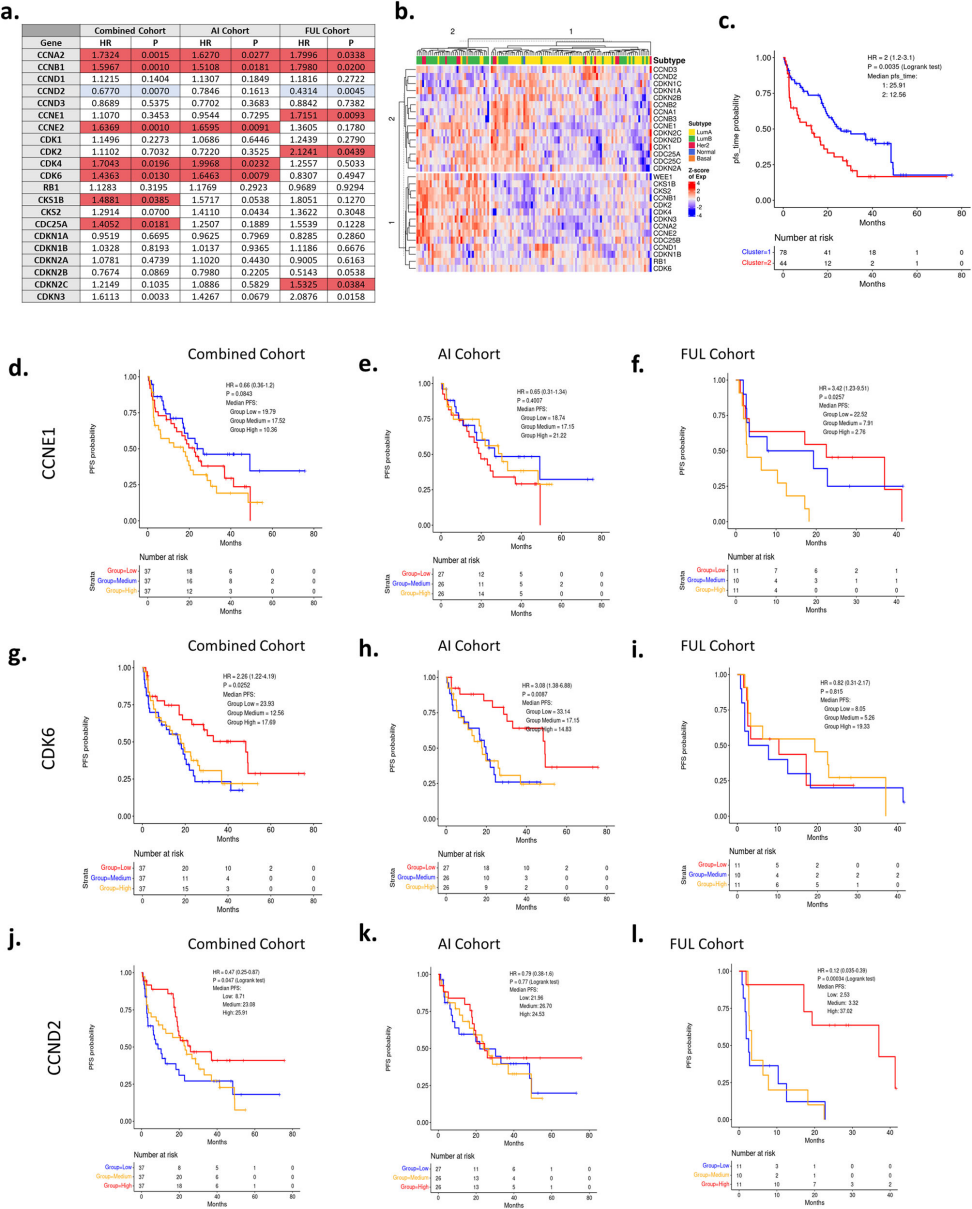
PFS with CDK regulatory genes. **a** Table summarizing association of PFS with major cell cycle genes across cohorts of patients. **b** Heatmap showing expression of these genes across pretreatment biopsies. This unsupervised clustering is able to stratify luminal A patients from other subtypes (mainly luminal B). **c** Kaplan–Meier plot showing progression-free survival difference between the two groups stratified by unsupervised clustering. *p* = 0.0035 by log rank test. **d**–**l** Testing for power of single genes in stratifying patients for progression-free survival using gene expression data from pretreatment biopsies. For CCNE1 in the combined, AI, and FUL cohorts, *p* = 0.0843, *p* = 0.4007, and *p* = 0.0257 by log rank test, respectively. For CDK6 in the combined, AI, and FUL cohorts, *p* = 0.0252, *p* = 0.0087, and *p* = 0.815 by log rank test, respectively. For CCND2 in the combined, AI, and FUL cohorts, *p* = 0.047, *p* = 0.77, and *p* < 0.001 by log rank test, respectively.

From preclinical studies, multiple genes associated with CDK regulation have been suggested to play a role in response to CDK4/6 inhibitors. Here we evaluated cell cycle related Cyclins and CDKs and key regulators (e.g., CDKN1B and CKS1B) in a relatively unbiased fashion. A subset of these genes was associated with shorter PFS (red), with only one gene associated with a longer PFS (blue) (Fig. 7a). Interestingly, a number of genes suspected to be associated with response (e.g., CCND1 and RB1) were not associated with PFS (Supplementary Fig. 7). Unsupervised hierarchical clustering illustrated that there is a degree of co-regulation of cell cycle genes, wherein high expression of many cell cycle genes is associated with shorter PFS (Fig. 7b, c). The behavior of CCNE1 and CDK6 are shown by Kaplan–Meier (K-M) plots in the combined cohort and treatment selective sub-cohorts (Fig. 7d–i). The only gene identified to associate with longer duration of PFS is CCND2 (Fig. 7j–l), which is inversely correlated with many of the other cell cycle regulated genes and is responsive to interferon/NF Kappa B signaling^46,47^.

### Biological features associated with PFS

**Fig. 8:**
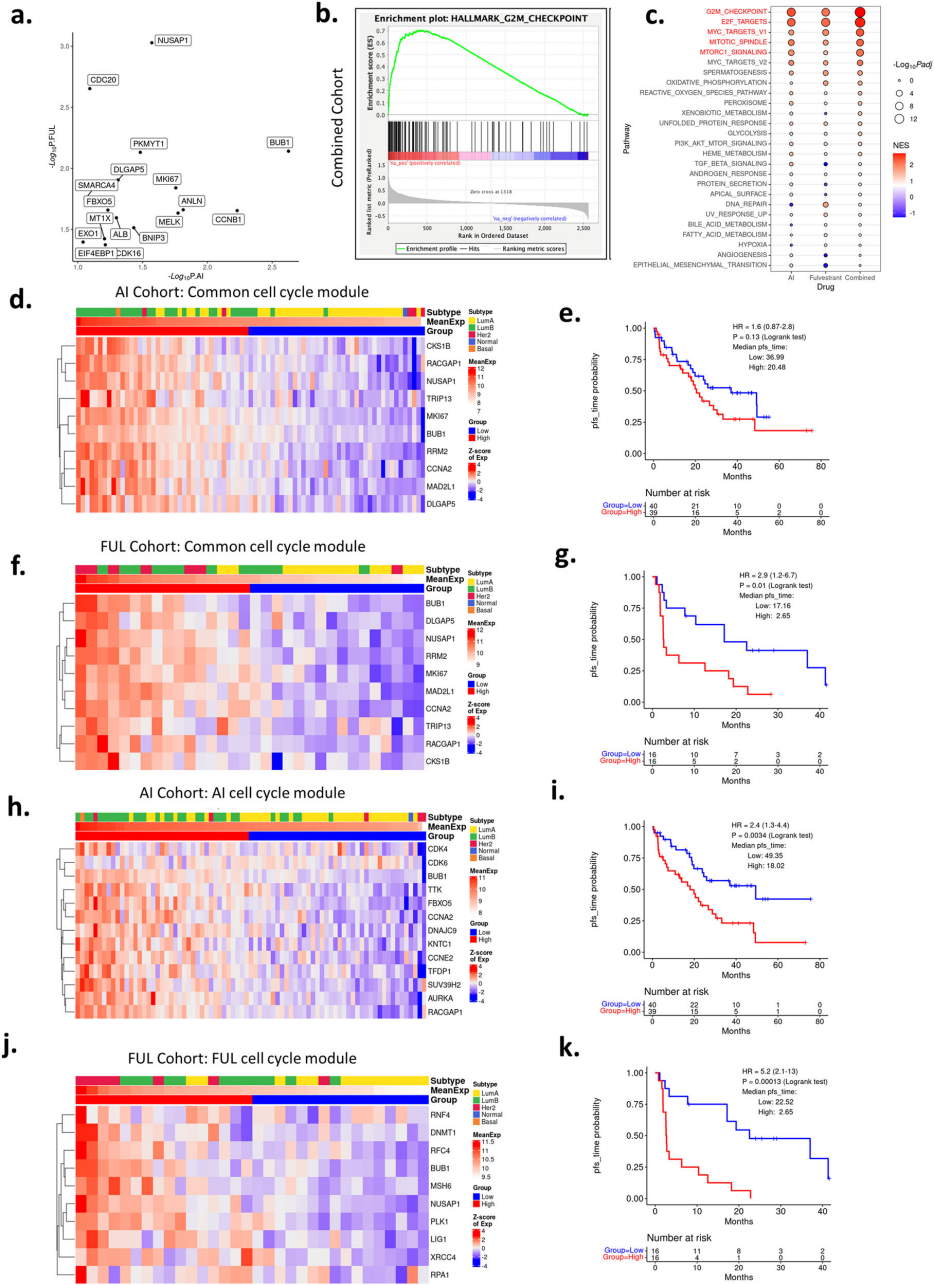
PFS with common cell cycle genes. **a** Genes associated with shorter PFS as identified by univariate Cox Proportional Hazards Regression on each of the treatment cohorts. −Log_10_ *p* values from the AI and the FUL cohort are shown on the *x*-axis and the *y*-axis, respectively. Data are filtered by HR < 1, *p* value ≤ 0.1 (AI cohort) or *p* value ≤ 0.05 (fulvestrant cohort). **b**, **c** Gene Set Enrichment Analysis for genes associated with shorter PFS. HR values from univariate Cox PH analysis on the combined cohorts were used to perform GSEA pre-ranked analysis. HR values are subtracted by 1 to center the genes with no association to zero. **d**–**g** Heatmap and K-M plots for common cell cycle gene modules tested on the AI (**d**, **e**) or FUL (**f**, **g**) treated cohort. *p* = 0.13 and *p* = 0.01, respectively, by log rank test. **h**–**k** Testing of cell cycle gene modules identified from each cohort separately for association with PFS on the same cohort. *p* = 0.003 and *p* < 0.001, respectively, by log rank test. Columns in these heatmaps are ordered by average log2-transformed normalized gene expression values from high (left) to low (right).

The data with PR, SBR and AIMS subtyping, suggest that there are intrinsic features associated with PFS in tumors treated with CDK4/6 inhibitors and endocrine therapy. To explore features associated with PFS duration, the cohort was divided according to the endocrine therapy (AI or FUL). For each therapy type, univariate Cox proportional hazard regression analysis was conducted to define genes that were independently associated (in terms of hazard ratios and *p* values) with sensitivity or resistance to the therapies (Supplementary Dataset 1). As shown in Fig. 8a, a collection of genes was associated with shorter PFS in both AI and FUL treatment groups. To determine the biological features associated with sensitivity or resistance, we employed ranked Gene Set Enrichment Analysis (GSEA) on the combined cohort^48^. In this analysis, genes are ranked on hazard ratios and tested for enrichment for the hallmark gene sets from the Molecular Signatures Database (MSigDB)^48,49^ on the high- and low-end of the HR spectrum. These analyses indicated that cell cycle genes regulated by RB/E2F were associated with short PFS in the combined cohort (Fig. 8a, b) as well as in each of the individual treatment groups (Fig. 8c). To explore predictive features of these gene sets, we utilized common cell cycle genes in the top 100 of genes associated with HR in both AI and FUL cohorts. This list of 10 genes was significantly associated with PFS in both the AI and the FUL cohorts (Fig. 8d–g). However, this combined gene set was inferior to the top cell cycle genes selective to either AI or FUL (Fig. 8h–k).

**Fig. 9:**
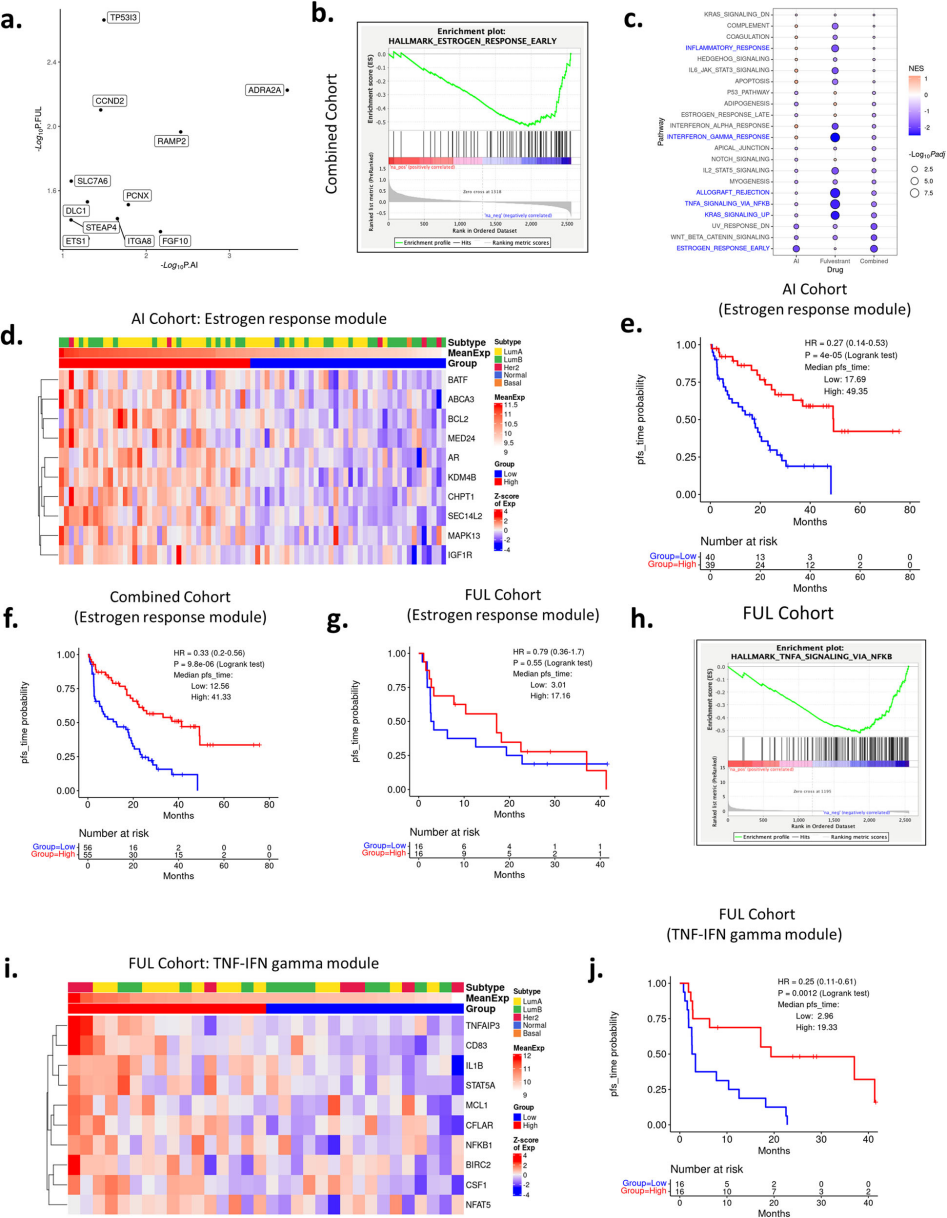
PFS with estrogen receptor and TNF/IFN gamma gene sets. **a** Genes associated with longer PFS as identified by univariate Cox Proportional Hazards Regression on each of the treatment cohorts. −Log_10_ *p* values from the AI and the FUL cohort are shown on the *x*-axis and the *y*-axis, respectively. Data are filtered by HR < 1, *p* value ≤ 0.1 (AI cohort) or *p* value ≤ 0.05 (fulvestrant cohort). **b** Estrogen response early gene set is associated with longer PFS as revealed by GSEA on combined cohort. **c** Bubble plot showing differential enrichment for gene sets between AI and fulvestrant treated cohorts. **d** Heatmap using genes from estrogen response module on gene expression data from pretreatment biopsies in the AI-treated cohort. **e** K-M plot showing PFS difference between high and low average expression values of this estrogen response gene module in the AI treated cohort. *p* < 0.001 by log rank test. **f**, **g** K-M plots showing survival difference between low and high average gene expression groups of the estrogen response gene module on the combined (**f**) or fulvestrant (**g**) treated cohorts. *p* < 0.001 and *p* = 0.55, respectively, by log rank test. **h** GSEA plot showing enrichment of genes in the TNF signaling via NFKB gene set. **i**, **j** Heatmap and K-M plot showing gene expression pattern of the TNF-IFN gamma gene module and its power in stratifying patients on the fulvestrant treated cohort using gene expression data from the pretreatment biopsies. *p* = 0.0012 by log rank test.

In terms of longer PFS, fewer common genes for AI and FUL treatment were observed for association (Fig. 9a). By GSEA analyses, estrogen receptor-signaling was the top gene set associated with longer PFS in the combined cohort, and the AI + CDK4/6 inhibitors group (Fig. 9b, c). A module of 10 estrogen receptor signaling associated genes was strongly associated with PFS in the AI cohort (Fig. 9d, e) and the combined cohort (Fig. 9f). In FUL + CDK4/6 inhibitor group, this pathway only trended toward longer PFS (Fig. 9g). Analyses of the FUL + CDK4/6 group using the same methodology showed that TNF signaling and interferon-gamma (IFN gamma) gene sets were associated with longer PFS (Fig. 9c, h). This association was confirmed in the context of clustering based on Euclidean distance and subsequent K-M analyses (Fig. 9i, j). Thus, while cell cycle signatures are commonly associated with shorter PFS, there are differential programs which appear dominant for AI vs FUL for longer PFS.

## Discussion

Here we used “real-world” clinical data and tissue samples to explore gene expression features of response to standard-of-care CDK4/6-inhibitor based therapy in HR+/HER2− metastatic breast cancer. To date, the only marker employed in delivering CDK4/6 inhibitors in this population is ER and HER2 status that is used to direct endocrine therapy, although potential features of response have been described from the analyses of the randomized clinical trials^26,38–40^.

Since in addition to ER, the status of PR was known, we could evaluate how PR status associates with the duration of PFS. In our cohort, which is composed of patients treated largely with palbociclib and AI or FUL, PR status was relevant in the AI treatment cohort. Our finding is consistent with another recently published real-world study that evaluated PR in a smaller cohort of patients^50^. Similar to our observations, they found that PR was not significant in the FUL treated cohort, suggesting that biological distinctions related to the endocrine sensitivity and prior treatments will have an impact on putative biomarkers. Nottingham scoring (modified SBR score) represents a composite of pathological features associated with tubular differentiation, nuclear pleomorphism and mitotic rate. As shown here, SBR scores were associated with the duration of PFS in our cohort. As with the PR, SBR showed significance only in the AI treated groups. These findings and work carried out with PALOMA-2 and PALOMA-3^39^, suggests that biomarkers developed will likely have to be selective for the line of treatment. Interestingly, a robust marker for the duration of PFS in the AI cohort was developed by simply combining the SBR and PR status. In the arm with SBR3 and PR low/deficient, the PFS was 6.21 months, suggesting that estimations of likely PFS could be deduced from a relatively small number of existing pathological measures in patients treated with CDK4/6 inhibitor and AI.

To expand beyond common clinical markers, we used the HTG Oncology biomarker panel which has been employed in multiple analyses from randomized clinical trials^23,26,38^. Using molecular subtyping (AIMS), we defined a degree of plasticity in gene expression during therapy. In both the remote recurrence and the metastatic setting prior to CDK4/6 inhibitor combination treatment, there were comparable frequencies of luminal A and luminal B tumors. During treatment, there is a shift toward luminal A and normal-like subtypes. This response profile is ostensibly due to the on-target effect of CDK4/6 inhibitors on the suppression of cell cycle genes that denote the predominant difference of luminal B as opposed to luminal A and normal like subtypes. Interestingly, investigating the behavior of tumors which “switched” to luminal A as opposed to originating from luminal A revealed that being luminal A prior to treatment had a longer PFS, although the numbers are too limited to make a robust conclusion (Supplementary Fig. 8). After disease progression, few luminal A tumors remained and there was a general further deregulation of cell cycle genes. These data suggest that while the therapy is effective at suppressing proliferation, once tumors progress, they have evolved to a more aggressive biology. This may also explain why in this cohort, post progression OS is essentially identical. In the context of the present study, the subtype of disease in the pretreatment biopsy was associated with duration of PFS. This largely followed the prognostic significance of breast cancer subtypes wherein luminal B, Her2, and basal are associated with poor outcome. Interestingly, this association of subtypes was only significant in the AI treated subgroup. Part of the lack of significance could be due to the relatively small number of patients in the FUL treated cohort, as well as the greater heterogeneity with regard to subtypes beyond luminal A and B.

To approach the biological features associated with PFS, a relatively unbiased approach was employed. Interestingly, it showed that cell cycle deregulation, exemplified by E2F target genes or G2/M associated genes are jointly associated with shortened PFS irrespective of the endocrine therapy employed, specifically when applied to the pretreatment biopsy (Supplementary Fig. 9). These findings are similar to those emerging from the analyses of the PALOMA-2/3 analyses^26,38–40^. However, at the gene-level there were differences between the treatment cohorts. Similarly, it would appear that CDK4/6 expression is relevant in the context of AI treated tumors, while CDK2 and CCNE1 are more relevant in the context of FUL treated tumors. Thus, whether the importance of select E2F-target genes is due to a primary mechanism of resistance e.g., CCNE1 vs CDK6 deregulation, remains unknown. In terms of longer PFS, estrogen receptor signaling was strongly associated with progression-free duration, most potently in the pretreatment biopsy as opposed to the primary/recurrence sample (Supplementary Fig. 9). This finding was also observed in analyses of PALOMA-2/3 studies^39^. However, in our FUL treated cohort there was minimal enrichment for these genes associated with duration of PFS. In contrast, the TNF and IFN-gamma gene sets were more strongly associated with longer PFS in FUL treated cohorts. This could relate also to the association of CCND2 with the duration of PFS predominantly in the FUL treatment group^47^. This finding contrasts with other settings, where it has been proposed that genes associated with interferon signaling are associated with resistance to CDK4/6 inhibitors in clinical populations^26^. The TNF and IFN-gamma gene sets are highly heterogeneous, and it will be important to evaluate individual genes between experimental groups to ultimately make strong conclusion. We believe that at least part of the signal being detected in the FUL cohort is reflective of TILs that are generally associated with improved prognosis in HR+/HER2− breast cancer. Immunologically restricted genes (e.g., CD40 and CD3D) were selectively associated with longer PFS in the FUL treated cohort (Supplementary Fig. 8). Interestingly, by using METABRIC data^51^, we could compare the association of the gene sets employed here with prognosis (Supplementary Fig. 10). Cell cycle modules are associated with prognosis, however, neither the estrogen nor the TNF gene modules were associated with disease-free survival in METABRIC samples. Surprisingly only the estrogen response module is strongly associated with OS in this cohort (Supplementary Fig. 11).

In total, the studies here identified multiple biological features that emerge during treatment with CDK4/6 inhibitors and suggested pathways relevant to PFS. However, further study will be required to develop these genes or clinical features to the point of a biomarker. Ongoing studies are developing independent cohorts to validate the findings here and to develop prospectively validated biomarkers for duration of response to CDK4/6 with endocrine therapy regimens.

## Methods

### Data source and patient selection

Chart review was conducted for 280 patients who were diagnosed with HR+/HER2− breast cancer and received a CDK4/6 inhibitor from 2015 to 2022 at Roswell Park Comprehensive Cancer Center as inclusion criteria for the study. The great majority (>95%) of consented patients were women. Patient data from two studies approved by the Roswell Park Comprehensive Cancer Center Institutional Review Board was used. A retrospective protocol was utilized to collect information and biospecimens on 71 patients. Subsequently a combination retrospective and prospective (NCT04526587) protocol was developed. Two hundred and twenty-nine patients have been consented as of December 22, 2022. Eligible patients were ≥18 years of age, had ER+/HER2− advanced breast cancer, and were treated with a CDK4/6 inhibitor. Electronic medical records were used to extract demographic information, smoking history, menopausal status, BMI, Eastern Cooperative Oncology Group (ECOG) performance status, surgery and pathology reports, genomic data, dates of diagnosis and recurrence(s), site(s) of metastases, and cancer treatment information.

### Ethics

The clinical information collected was approved by the Institutional Research Board of Roswell Park Cancer Center. Written informed consent was obtained for all patients through either the clinical trial (NCT04526587) or the Roswell Park Remnant Tissue protocol and corresponding IRB approved investigator protocol specific to this study.

### Tissue selection

All surgical pathology case slides from the Roswell Park Comprehensive Cancer Center Department of Pathology were reviewed for each patient to ensure adequate tumor tissue. Chosen cases were then coded by specific timepoint during their treatment as shown in Fig. 4b. Selected blocks were sectioned, and regions of high tumor cellularity were defined by a breast pathologist (AKW). Slides were then sent to HTG Molecular Diagnostics, Inc. for targeted gene expression consistent with other studies^26,39,40^.

### Statistical analysis

The PFS was defined as the time from the first dose of CDK4/6i inhibitor therapy to either scan- or marker-proven progression, or death while on therapy. The OS, was defined as the time of the biopsy (or scan if no biopsy was performed) leading to the initiation of CDK4/6 inhibitor therapy, to death or date of last follow-up. Patients who stopped ciclib therapy due to toxicities (*n* = 22) were not considered in PFS calculations. For the primary purpose of this study, patients were divided into 2 groups: patients taking an aromatase inhibitor (letrozole, anastrozole, or exemestane) with a CDK 4/6 inhibitor, and patients taking fulvestrant with a CDK 4/6 inhibitor. K-M survival analysis compared with log-rank tests, univariate, and multivariate Cox proportional-hazards regression were used to compare PFS by endocrine therapy. R version 3.6.1 or 4.2.0 (R Foundation for Statistical Computing, Vienna, Austria) was used for all statistical analyses.

### RNA-Seq data preparation and processing

Patient tissue samples that were obtained during standard-of-care from various clinical timepoints were sent to HTG Molecular Diagnostics, Inc. for targeted sequencing using their HTG EdgeSeq Oncology Biomarker Panel consisting of 2549 selected cancer associated genes. Raw data was assembled from four separate HTG runs. Raw read counts from four batches were subjected to batch effect removal using ComBat-seq^52^. The batch effect corrected raw read count matrix is then used as input to edgeR^53^ for data normalization. Normalized data was then log2 transformed by adding a pseudo-count of 1 to each value in the data matrix and subsequently used for downstream analyses.

### Paired sample differential gene expression analysis

Differential gene expression analysis was performed between different timepoints (on-treatment vs pretreatment, post-treatment vs pretreatment, pretreatment vs primary/recurrence) on paired patient specimens using the DESeq2^54^ (v1.36.0) Bioconductor package.

### Gene Set Enrichment Analysis (GSEA)

We used a locally installed GSEA software (v4.2.1) to perform GSEA Preranked analyses to identify associations of gene sets in the HALLMARK dataset in the Molecular Signature Database (h.all.v2023) with gene lists ranked by hazard ratios (substracted by one, to center the HR = 1 (no association) to zero) (Figs. 8b and 9b, h) or with magnitude (Log2FC) of differentially expressed genes between timepoints (Fig. 4d). To obtain normalized enrichment score and *p* values for generating plots in Figs. 4c, 8c, and 9c, we used the fgsea Bioconductor package (10.1101/060012) and custom R code.

### External datasets used in this study

The NeoPalAna dataset was retrieved from Gene Expression Omnibus (GEO) with accession number GSE93204. We used the GEOquery (v2.64.2) Bioconductor package for retrieving the gene expression data and clinical information. The METABRIC dataset^51^ was downloaded from cBioPortal. To match patient characteristics, we extracted a subset of this dataset to include only those patients with HR+/HER2− status.

### Intrinsic subtype assignment

To predict intrinsic cancer subtypes, we used the AIMS^45^ R package. Since the HTG gene panel does not include all the genes used in AIMS, we evaluated subtype classification accuracy with the reduced gene set used in the HTG panel. We first ran the example code in the AIMS documentation with data included in that package. Next, we ran the code again with the reduced testing dataset by including only genes available in the HTG gene panel. This resulted in the misclassification of 52 samples out of the 321 samples in total (an 84% of concordance). To run AIMS, we replaced the gene symbol in the raw RNA-Seq read count data matrix with the Entrez gene ID using the org.Hs.eg.db Bioconductor R package (v3.10.0).

### Gene module selection

Univariate Cox regression analysis were performed on each of the treatment cohorts (AI, fulvestrant, and combined) separately, using the gene expression data from pretreatment biopsies. The hazard ratio values were subtracted by 1 to have zero values indicating no association (HR = 1) and used in GSEA pre-ranked analysis against the HALLMARK gene sets (h.all.v2023). We identified the E2F Targets and G2M Checkpoint as the top enriched gene sets, Estrogen Response Early as the top depleted gene set in the AI treated cohort, IFN-gamma response and TNF signaling via NFKB as the top depleted gene sets in the fulvestrant treated cohort. The leading-edge genes in each of the top enriched/depleted gene sets are intersected with the top 100 high HR genes or the bottom 100 low HR genes. We plotted heatmaps for these genes sets and selected ten genes based on their expression pattern. We termed them as “Estrogen Response Module” for the 10 genes selected using the AI-treated cohort, “TNF-IFN gamma Module” for the 10 genes selected using the fulvestrant-treated cohort, and “Common Cell Cycle Module” for the ten genes selected from both the AI and the fulvestrant combined cohorts.

### Plotting

The Sankey plot was made using the plotly R package (v4.9). We first performed prediction of tumor intrinsic subtypes for all samples using AIMS. The prediction results for samples from the same patient with different timepoints (primary, pretreatment, on-treatment, and post-treatment) were used as input to a custom R script to create the Sankey plot. All the heatmaps are potted using the ComplexHeatmap^55^ R package (v2.12.1). K-M plots are based on the survival R package (3.4–0) with custom wrapper code for enhancements. Other plots were prepared using the ggplot2 (v3.3.6) or ggpubr (v0.4.0) R packages.

### mIF staining

Formalin-fixed paraffin-embedded (FFPE) 4 µm sections were cut and placed on charged slides. Slides were dried at 65 °C for 2 h. After drying, the slides were placed on the BOND RX^m^ Research Stainer (Leica Biosystems) and deparaffinized with BOND Dewax solution (AR9222, Lecia Biosystems). The multispectral immunofluorescent (mIF) staining process involved serial repetitions of the following for each biomarker: epitope retrieval/stripping with ER1 (citrate buffer pH 6, AR996, Leica Biosystems) or ER2 (Tris-EDTA buffer pH9, AR9640, Leica Biosystems), blocking buffer (AKOYA Biosciences), primary antibody, Opal Polymer HRP secondary antibody (AKOYA Biosciences), Opal Fluorophore (AKOYA Biosciences). All AKOYA reagents used for mIF staining come as a kit (NEL821001KT). Spectral DAPI (AKOYA Biosciences) was applied once slides were removed from the BOND. They were cover slipped using an aqueous method and Diamond antifade mounting medium (Invitrogen ThermoFisher). The mIF panel consisted of the following antibodies: Ki67 (Abcam, ab16667), AE1AE3 (Dako, M3515), CCNE (Abcam, ab33911), CCND1 (ThermoFisher, MA1-39546), CCNA (Abcam, ab32386), RB (Cell Signaling, 9309s), pRB (Cell Signaling, 8516), and MCM2 (BioSb, BSB6334).

### Tissue imaging and analysis

Slides were imaged on the PhenoImager™ HT (AKOYA Biosciences). Further analysis of the slides was performed using inForm® Software v2.6.0 (AKOYA Biosciences). The whole slides were first scanned in an unmixed view, then representative ROIs were selected for acquisition under guidance of a pathologist. These ROIs were then rescanned to achieve full spectral unmixing. A representative subset of these unmixed ROIs was then used to train tissue and cell segmentation. Next a unique algorithm was created using a machine learning technique, in which the operator selects positive and negative cell examples for each marker. These algorithms were then batch applied across a greater number of ROIs selected for inclusion in further analysis. The RStudio plugin, phenoptrReports, was used to extract phenotype counts from the resulting data tables.

### Reporting summary

Further information on research design is available in the Nature Research Reporting Summary linked to this article.

### Supplementary information

Supplementary Figures

Reporting Summary

Supplementary Dataset 1

## Data Availability

The datasets generated and analyzed in the current study are available from the corresponding author on reasonable request.
