# Supplementary material for: Determinants of response to CDK4/6 inhibitors in the real-world setting
Source: NPJ Precis Oncol. 2023 Sep 13;7:90. doi: 10.1038/s41698-023-00438-0 (PMC10499925; doi:10.1038/s41698-023-00438-0)
Supplement: Supplementary file 1 — Supplementary Figures [file 41698_2023_438_MOESM1_ESM.pdf]

a.

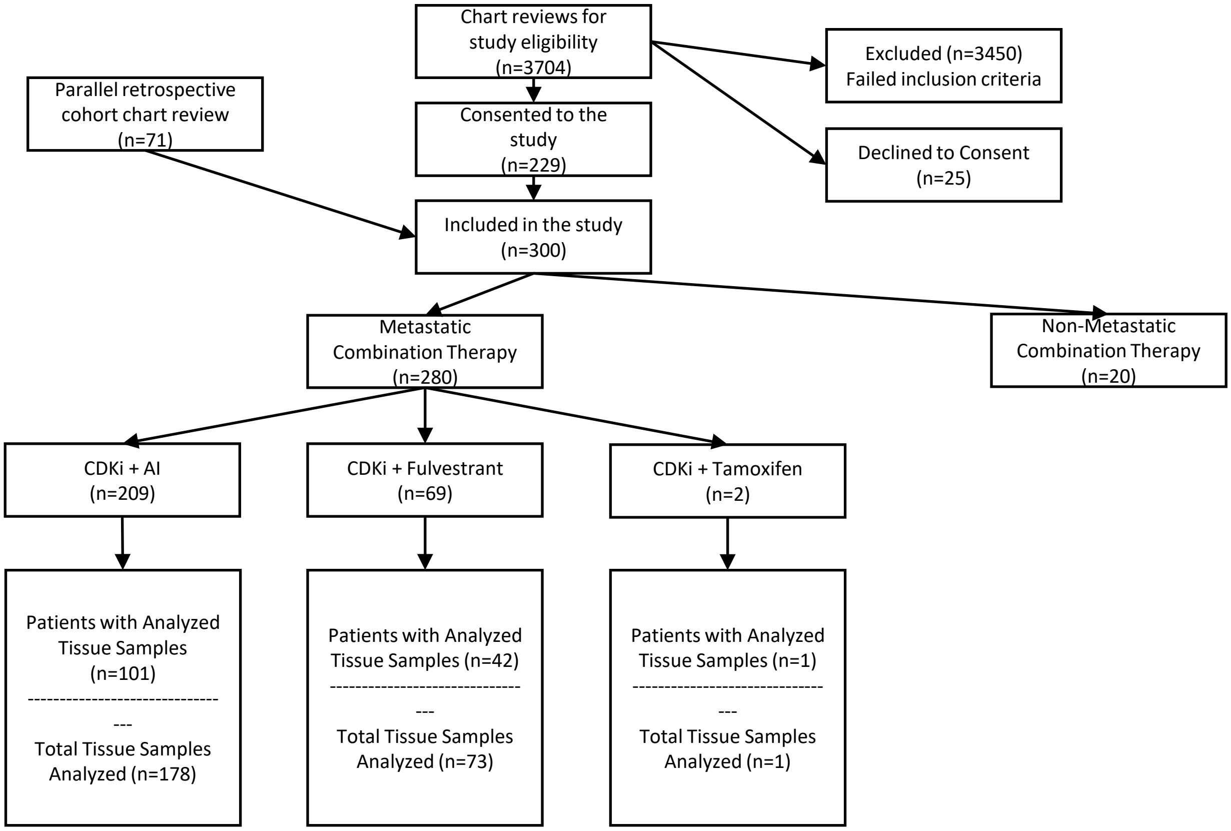


b.

| | Combined | | AI | | FUL | |
|---|---|---|---|---|---|---|
| Variable | HR | P | HR | P | HR | P |
| **Visceral Status** | 1.4196 | 0.0331 | 1.324 | 0.164 | 1.3728 | 0.281 |
| **Prior Endocrine Therapy** | 1.9834 | 0.00041 | 1.7144 | 0.0123 | 2.2928 | 0.165 |
| **De Novo vs Recurrent Disease** | 0.7323 | 0.0929 | 0.7458 | 0.17 | 1.3495 | 0.468 |

**c.**

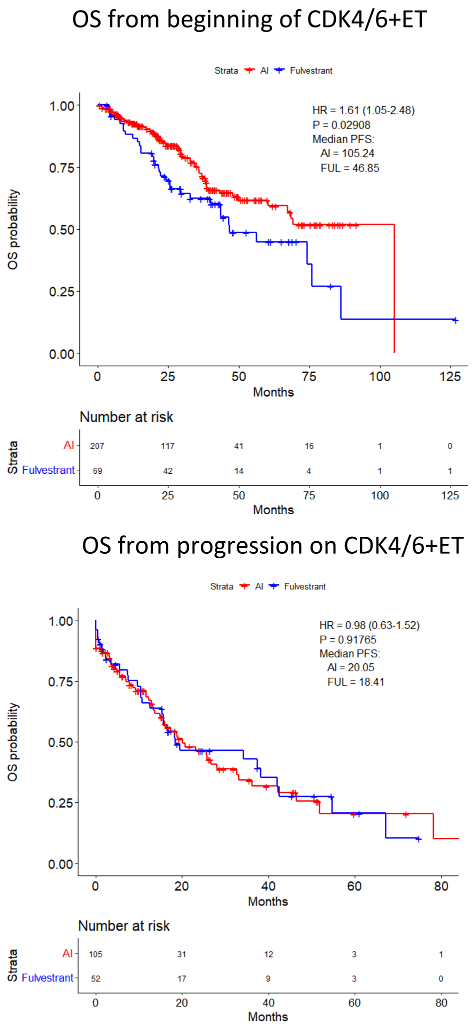


**d.**

| Treatment | Freq | % | Cumulative % |
|---|---|---|---|
| Xeloda | 89 | 18.23 | 18.2 |
| Taxol | 56 | 11.5 | 29.7 |
| Exemestane + Everolimus | 41 | 8.4 | 38.0 |
| Eribulin | 32 | 6.5 | 52.1 |
| Fulvestrant + Piqray | 29 | 5.9 | 58.1 |
| Doxil | 23 | 4.7 | 62.8 |
| Fulvestrant + Everolimus | 19 | 3.9 | 66.7 |
| Olaparib | 15 | 3.1 | 69.7 |
| Enhertu | 15 | 3.1 | 72.8 |
| Sacituzumab | 12 | 2.5 | 75.3 |
| Anastrozole | 11 | 2.2 | 77.5 |
| Abraxane | 10 | 2 | 79.6 |
| Taxol + Gemcitabine | 9 | 1.8 | 81.4 |
| Letrozole | 9 | 1.8 | 83.2 |
| Exemestane | 8 | 1.6 | 84.9 |
| Adriamycin + Cytoxan | 8 | 1.6 | 86.5 |
| Ixabepoline | 7 | 1.4 | 87.9 |
| Carboplatin + Gemcitabine | 7 | 1.4 | 89.4 |
| Gemcitabine | 5 | 1 | 90.4 |
| Vinorelbine | 4 | 0.8 | 91.2 |
| Palbociclib + Fulvestrant | 4 | 0.8 | 92.0 |

**Supplementary Figure 1**. (a) CONSORT diagram displaying patient screening and selection for gene expression analysis. (b) Table summarizing association of progression free survival with clinically significant variables across the cohorts of patients. (c) Kaplan-Meier analysis of overall survival from start of CDK4/6-based therapy and progression on CDK4/6-based therapy between patients taking AI vs Fulvestrant. p=0.029 and p=0.918 respectively. (d) Table of the predominant therapies after progression on CDK4/6-based therapy.

AI Cohort (HER2)

**a.**

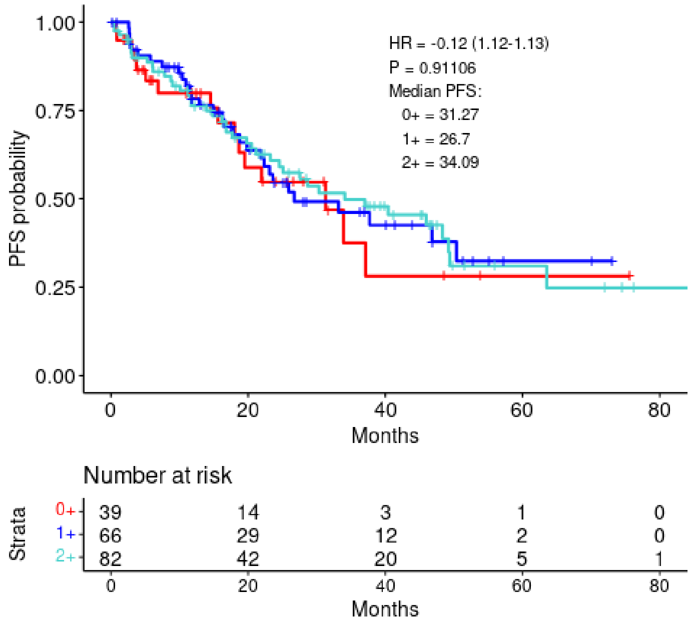


FUL Cohort (HER2)

**b.**

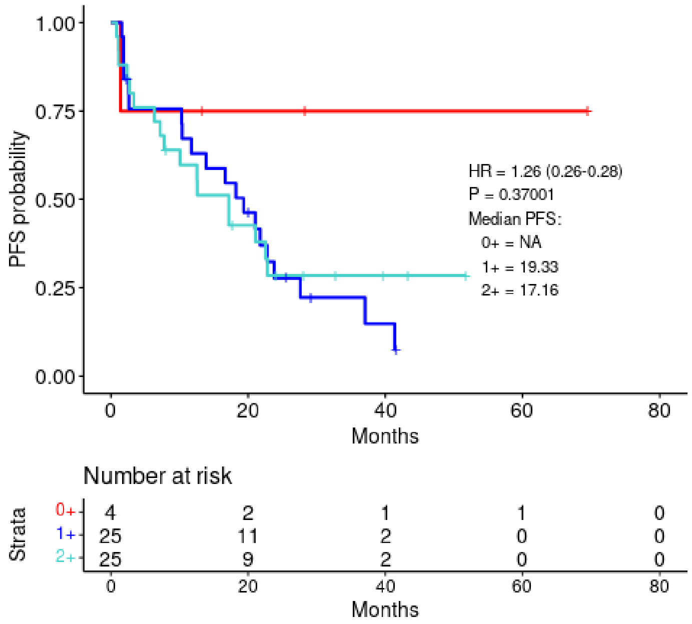


**Supplementary Figure 2.** (a) Kaplan-Meier analysis of progression free survival comparing HER2 expression by IHC across patients taking AI. p=0.911 by log-rank. (b) Kaplan-Meier analysis of progression free survival comparing HER2 expression by IHC across patients taking Fulvestrant. p=0.37 by log-rank.

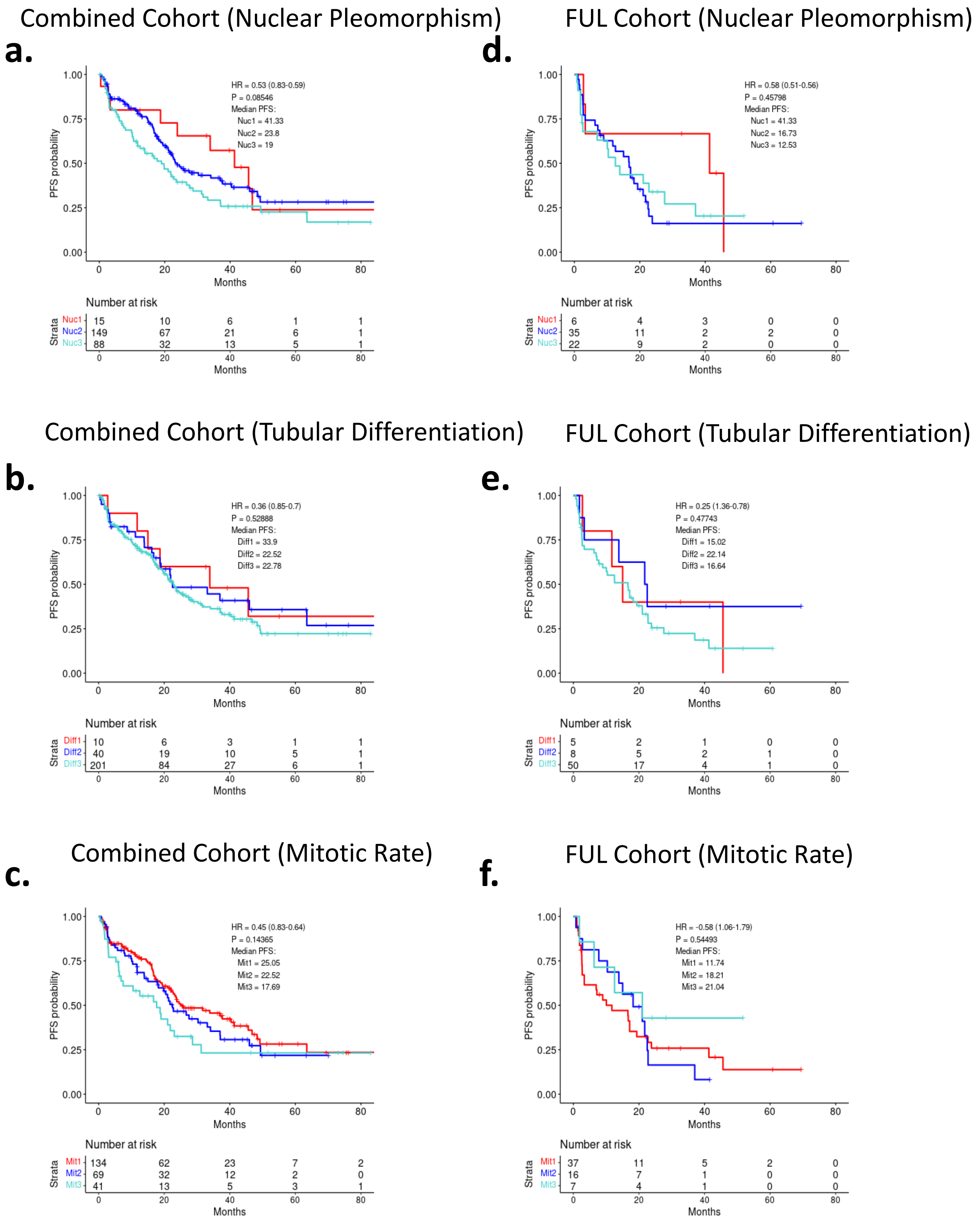


**Supplementary Figure 3.** (a) Kaplan-Meier analysis of progression free survival comparing Nuclear Pleomorphism across all patients. p=0.085 by log-rank. (b) Kaplan-Meier analysis of progression free survival comparing Tubular Differentiation across all patients. p=0.529 by log-rank. (c) Kaplan-Meier analysis of progression free survival comparing Mitotic Rate across all patients. p=0.144 by log-rank. (d) Kaplan-Meier analysis of progression free survival comparing Nuclear Pleomorphism across patients taking Fulvestrant. p=0.458 by log-rank. (e) Kaplan-Meier analysis of progression free survival comparing Tubular Differentiation across patients taking Fulvestrant. p=0.477 by log-rank. (f) Kaplan-Meier analysis of progression free survival comparing Mitotic Rate across patients taking Fulvestrant. p=0.545 by log-rank.

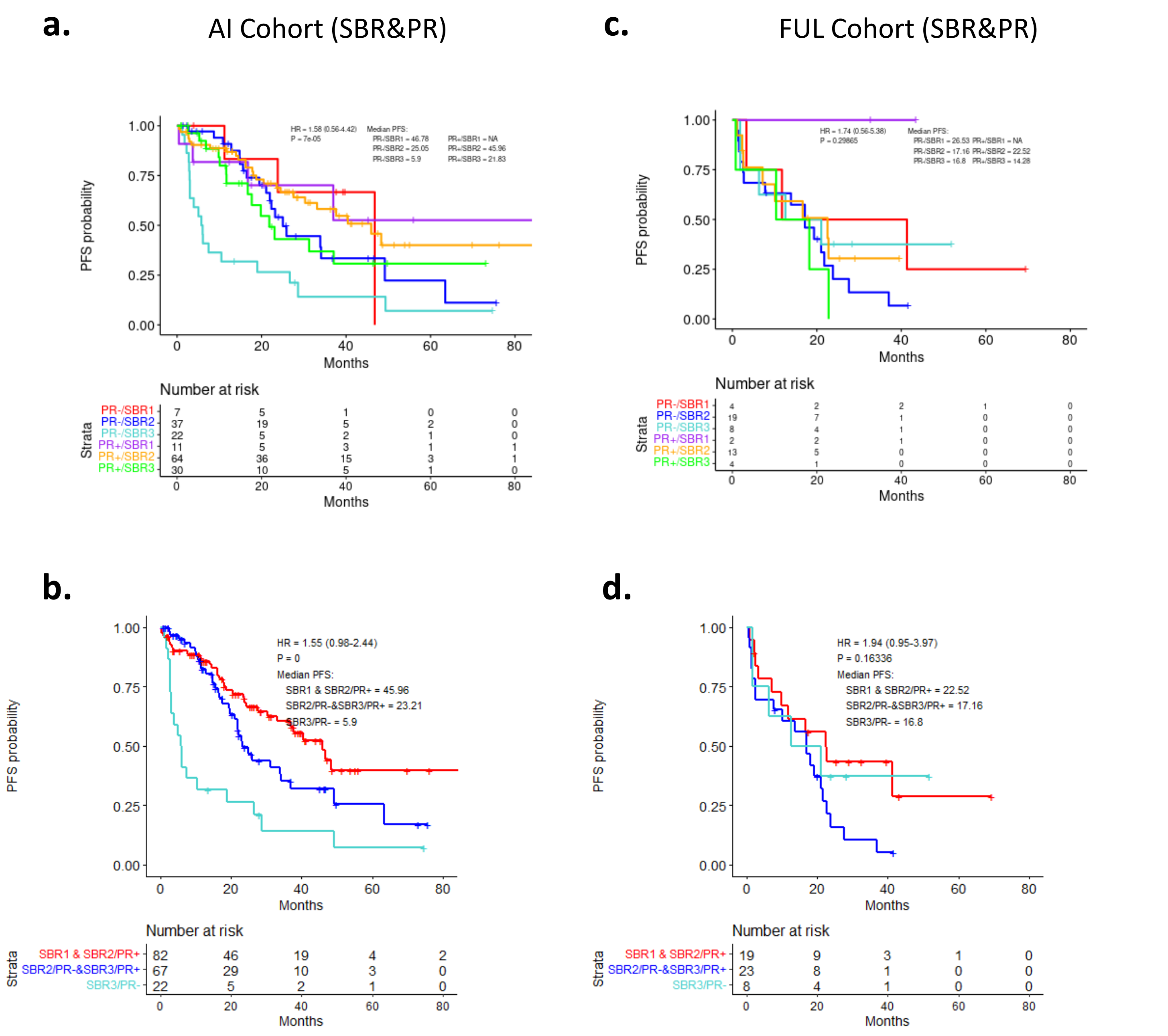


**Supplementary Figure 4.** (a) Kaplan-Meier analysis of progression free survival comparing combinations of overall SBR score and PR status across patients taking AI. $p<0.001$ by log-rank. (b) Kaplan-Meier analysis of progression free survival comparing subgroups of overall SBR score and PR status across patients taking AI. $p<0.001$ by log-rank. (c) Kaplan-Meier analysis of progression free survival comparing combinations of overall SBR score and PR status across patients taking Fulvestrant. $p=0.299$ by log-rank. (d) Kaplan-Meier analysis of progression free survival comparing subgroups of overall SBR score and PR status across patients taking Fulvestrant. $p=0.163$ by log-rank.

**a.**

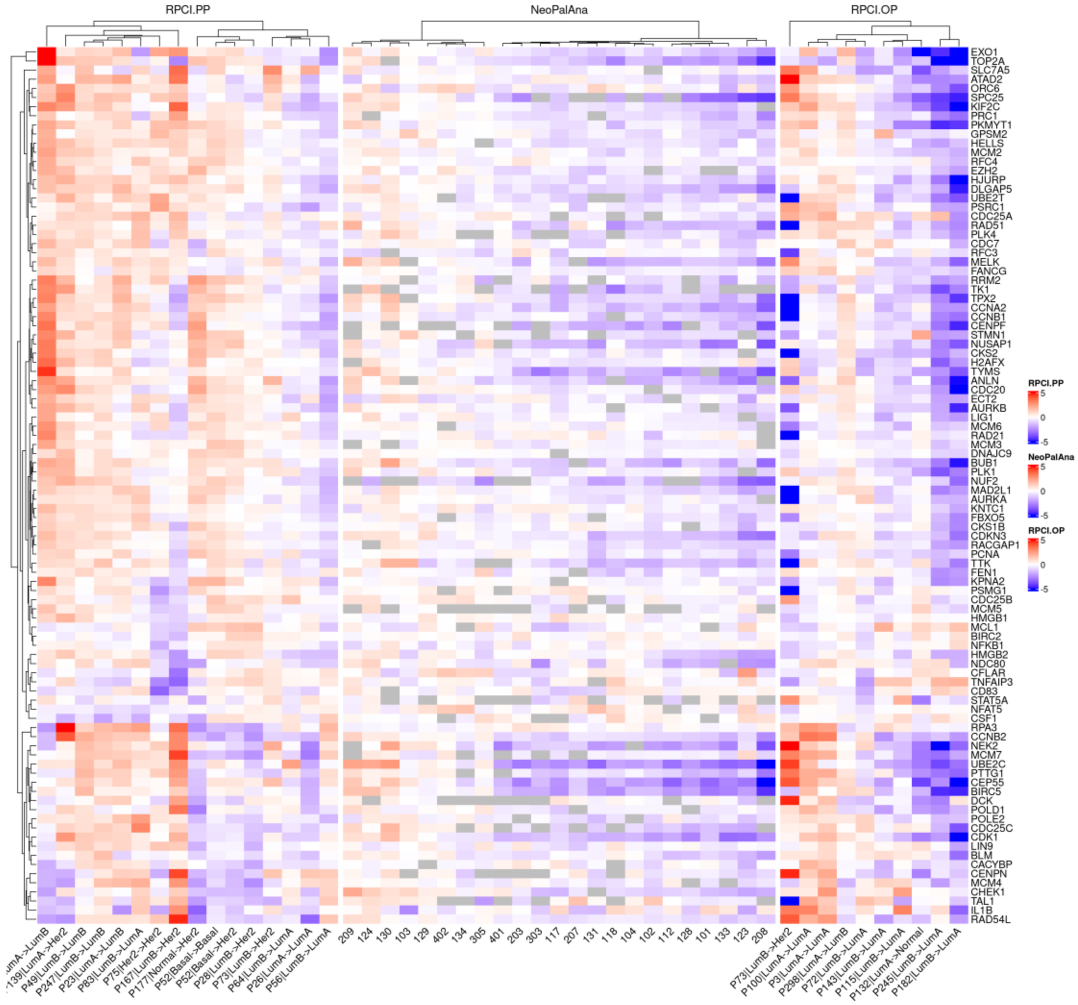

RPCI.PP
NeoPalAna
RPCI.OP
EXO1
TOP2A
SLC7A5
ATAD2
ORC6
SPC25
KIF2C
PRC1
PKMYT1
GPSM2
HELLS
MCM2
RFC4
EZH2
HJURP
DLGAP5
UBE2T
PSRC1
CDC25A
RAD51
PLK4
CDC7
RFC3
MELK
FANCG
RRM2
TK1
TPX2
CCNA2
CCNB1
CENPF
STMN1
NUSAP1
CKS2
H2AFX
TYMS
ANLN
CDC20
ECT2
AURKB
LIG1
MCM6
RAD21
MCM3
DNAJC9
BUB1
PLK1
NUF2
MAD2L1
AURKA
KNTC1
FBXO5
CKS1B
CDKN3
RACGAP1
PCNA
TTK
FEN1
KPNA2
PSMG1
CDC25B
MCM5
HMGB1
MCL1
BIRC2
NFKB1
HMGB2
NDC80
CFLAR
TNFAIP3
CD83
STAT5A
NFAT5
CSF1
RPA3
CCNB2
NEK2
MCM7
UBE2C
PTTG1
CEP55
BIRC5
DCK
POLD1
POLE2
CDC25C
CDK1
LIN9
BLM
CACYBP
CENPN
MCM4
CHEK1
TAL1
IL1B
RAD54L
RPCI.PP
5
0
-5
NeoPalAna
5
0
-5
RPCI.OP
5
0
-5
P49|LumB->LumB
P247|LumB->LumB
P23|LumA->LumB
P83|LumB->LumA
P75|Her2->Her2
P167|LumB->Her2
P177|Normal->Her2
P52|Basal->Basal
P52|Basal->Her2
P28|LumB->Her2
P73|LumB->Her2
P64|LumB->LumA
P26|LumA->LumA
P56|LumB->LumA
209
124
130
103
129
402
134
305
401
203
303
117
207
131
118
104
102
112
128
101
133
123
208
P73|LumB->Her2
P100|LumA->LumA
P3|LumA->LumA
P298|LumA->LumB
P72|LumB->LumA
P143|LumB->LumA
P115|LumB->LumA
P132|LumA->Normal
P245|LumB->LumA
P182|LumB->LumA

**b.**

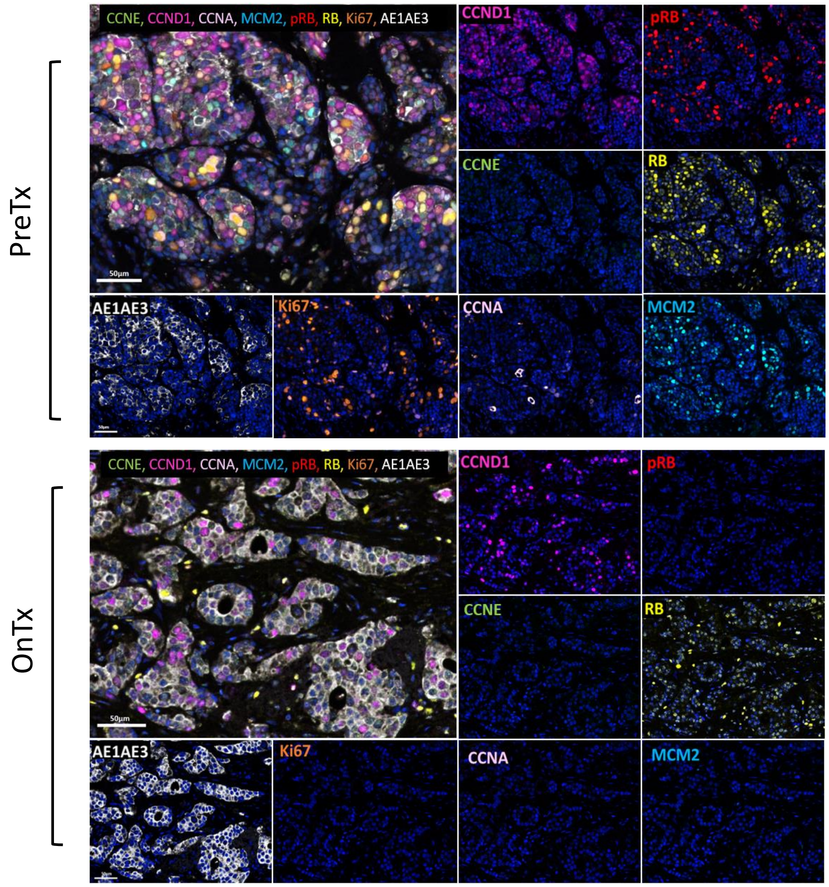


**Supplementary Figure 5.** (a). Heatmap showing suppression of cell cycle gene expression comparing pre- and on-treatment samples, and pre-treatment and post-progression samples (left and right portion) with the data from the NeoPalAna study (center). (b) Multispectral immunofluorescence imaging of paired pre-treatment and on-treatment samples showing suppression of select cell cycle proteins.

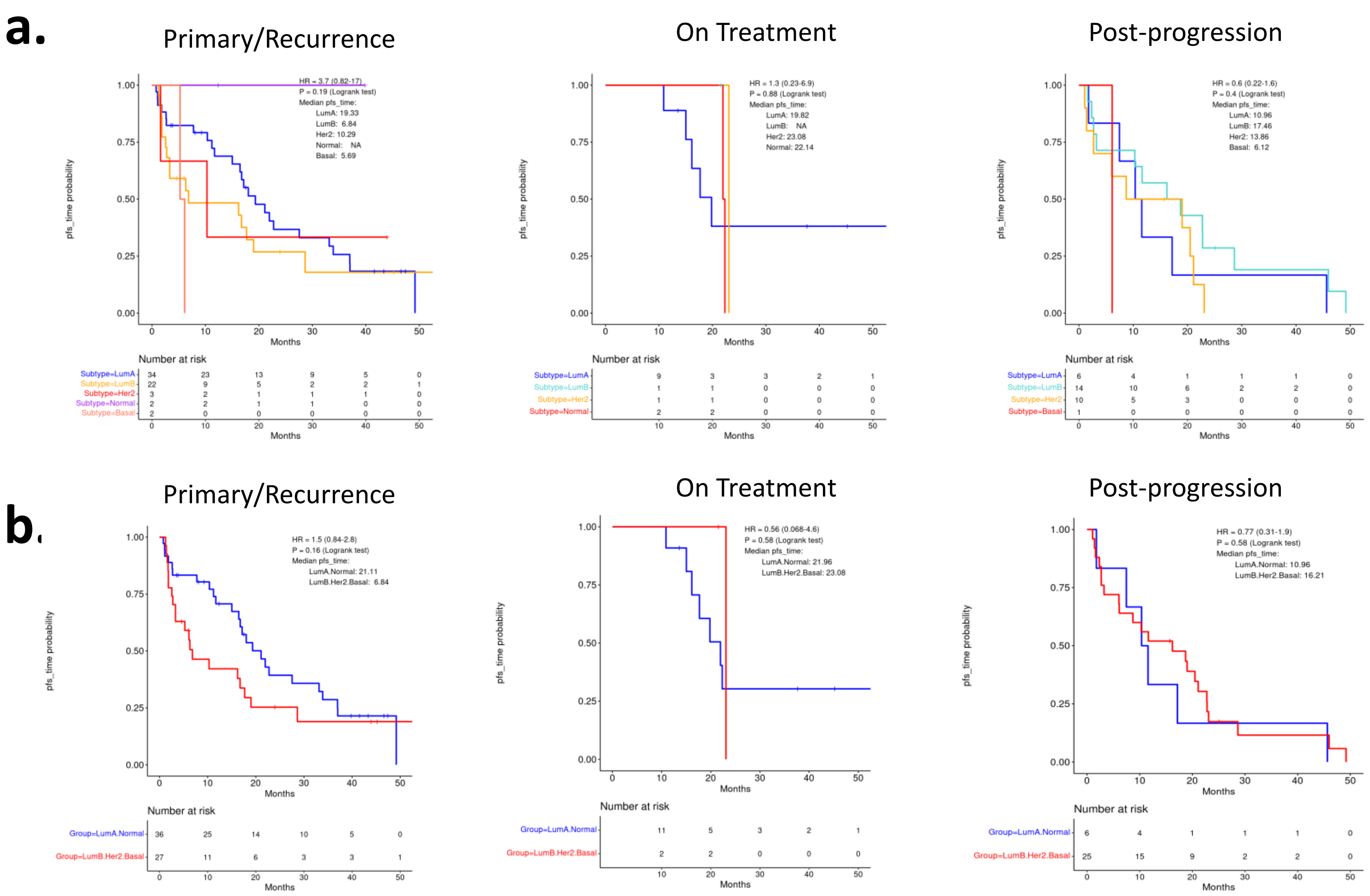
a.
Primary/Recurrence
HR = 3.7 (0.82-17)
P = 0.19 (Logrank test)
Median pfs_time:
LumA: 19.33
LumB: 6.84
Her2: 10.29
Normal: NA
Basal: 5.69
pfs_time probability
1.00
0.75
0.50
0.25
0.00
0 10 20 30 40 50
Months
Number at risk
Subtype=LumA 34 23 13 9 5 0
Subtype=LumB 22 9 5 2 2 1
Subtype=Her2 3 2 1 1 1 0
Subtype=Normal 2 2 1 1 0 0
Subtype=Basal 2 0 0 0 0 0
Months
On Treatment
HR = 1.3 (0.23-6.9)
P = 0.88 (Logrank test)
Median pfs_time:
LumA: 19.82
LumB: NA
Her2: 23.08
Normal: 22.14
pfs_time probability
Months
Number at risk
Subtype=LumA 9 3 3 2 1
Subtype=LumB 1 1 0 0 0
Subtype=Her2 1 1 0 0 0
Subtype=Normal 2 2 0 0 0
Months
Post-progression
HR = 0.6 (0.22-1.6)
P = 0.4 (Logrank test)
Median pfs_time:
LumA: 10.96
LumB: 17.46
Her2: 13.86
Basal: 6.12
pfs_time probability
Months
Number at risk
Subtype=LumA 6 4 1 1 1 0
Subtype=LumB 14 10 6 2 2 0
Subtype=Her2 10 5 3 0 0 0
Subtype=Basal 1 0 0 0 0 0
Months
b.
Primary/Recurrence
HR = 1.5 (0.84-2.8)
P = 0.16 (Logrank test)
Median pfs_time:
LumA.Normal: 21.11
LumB.Her2.Basal: 6.84
pfs_time probability
Months
Number at risk
Group=LumA.Normal 36 25 14 10 5 0
Group=LumB.Her2.Basal 27 11 6 3 3 1
Months
On Treatment
HR = 0.56 (0.068-4.6)
P = 0.58 (Logrank test)
Median pfs_time:
LumA.Normal: 21.96
LumB.Her2.Basal: 23.08
pfs_time probability
Months
Number at risk
Group=LumA.Normal 11 5 3 2 1
Group=LumB.Her2.Basal 2 2 0 0 0
Months
Post-progression
HR = 0.77 (0.31-1.9)
P = 0.58 (Logrank test)
Median pfs_time:
LumA.Normal: 10.96
LumB.Her2.Basal: 16.21
pfs_time probability
Months
Number at risk
Group=LumA.Normal 6 4 1 1 1 0
Group=LumB.Her2.Basal 25 15 9 2 2 0
Months

**c.**

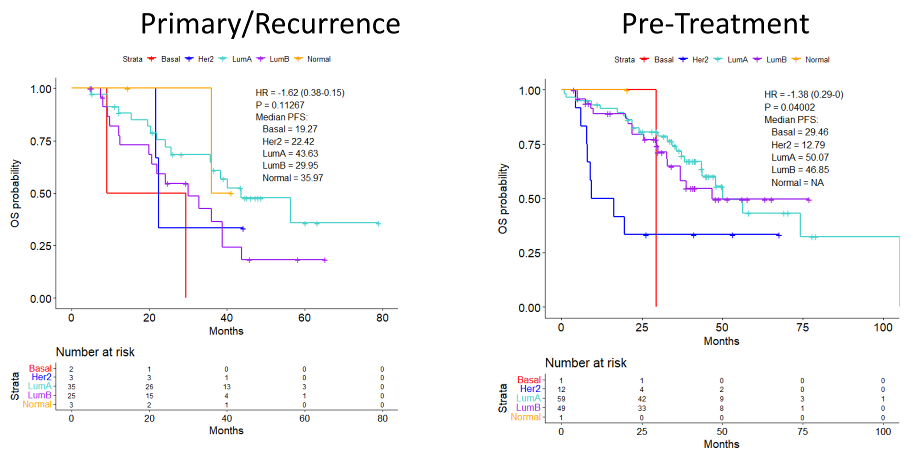


**d.**

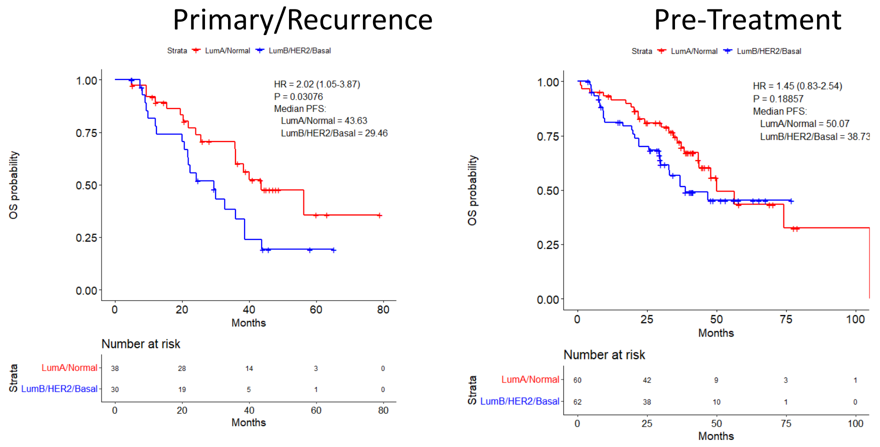


**Supplementary Figure 6.** Testing of survival difference between AIMS subtypes predicted using gene expression data at different treatment timepoints as indicated. (a) Progression-free survival of all AIMS subtypes for primary/recurrence, on treatment, and post-progression timepoints. p=0.19, p=0.88, and p=0.4 by log-rank, respectively. (b) Progression-free survival of LuminalA/Normal and LuminalB/HER2/Basal subtypes for primary/recurrence, on treatment, and post-progression timepoints. p=0.16, p=0.58, and p=0.58 by log-rank, respectively. (c) Overall survival of all AIMS subtypes for primary/recurrence and pre-treatment timepoints. p=0.1127 and p=0.04 by log-rank, respectively. (d) Overall survival of LuminalA/Normal and LuminalB/HER2/Basal subtypes for primary/recurrence and pre-treatment timepoints. p=0.0308 and p=0.1886 by log-rank, respectively.

Supplementary Figure 7

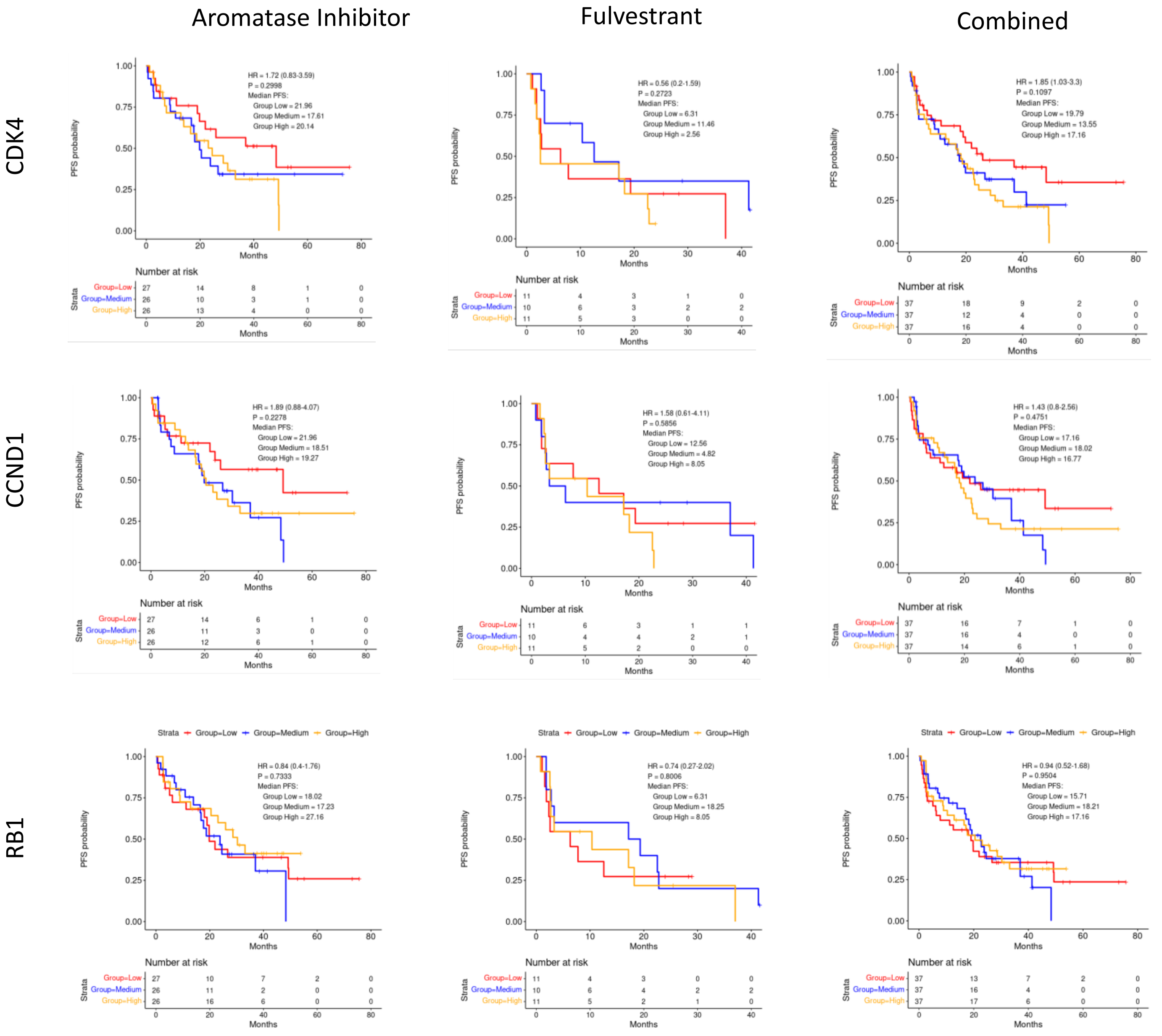


**Supplementary Figure 7.** Testing for association of single genes with progression free survival on the AI, Fulvestrant or combined cohorts. Patients are stratified by tertile of each of the single gene expression values. For CDK4, p=0.2998, p=0.2723, and p=0.1097 by log-rank, respectively. For CCND1, p=0.2278, p=0.5856, and p=0.4751 by log-rank, respectively. For RB1, p=0.7333, p=0.8006, and p=0.9504 by log-rank, respectively.

**a.**

| | Combined Cohort | | AI Cohort | | FUL Cohort | |
|---|---|---|---|---|---|---|
| **Gene** | **HR** | **P** | **HR** | **P** | **HR** | **P** |
| **CD22** | 0.7976 | 0.0214 | 0.7999 | 0.0598 | 0.8108 | 0.2675 |
| **CD70** | 0.8529 | 0.0106 | 0.8481 | 0.0257 | 0.8032 | 0.1109 |
| **CD79A** | 0.8433 | 0.0108 | 0.8729 | 0.0880 | 0.6689 | 0.0283 |
| **CD83** | 1.0239 | 0.7442 | 1.0944 | 0.3833 | 0.6689 | 0.0297 |
| **BATF** | 0.6229 | 0.0008 | 0.5185 | 0.0004 | 0.8692 | 0.5042 |
| **CD3D** | 0.8627 | 0.0774 | 0.9094 | 0.3457 | 0.6449 | 0.0137 |
| **CD27** | 0.8457 | 0.0061 | 0.8786 | 0.0755 | 0.7625 | 0.0424 |
| **CD5** | 0.8791 | 0.0645 | 0.9123 | 0.2809 | 0.7459 | 0.0371 |

**b.**

| Subtype | P-code | PFS time | Average PFS |
|---|---|---|---|
| **LumA to LumA** | P100 | 37.64342309 | 41.4242 |
| | P3 | 45.20498405 | |
| **LumB to Her2** | P73 | 23.07919913 | 23.0792 |
| **LumB to LumA** | P115 | 10.88207253 | 23.1515 |
| | P143 | 17.6874774 | |
| | P182 | 19.82444028 | |
| | P245 | 13.54505704 | |
| | P72 | 53.81858829 | |

**Supplementary Figure 8.** (a) Table summarizing association of PFS with select immune gene expression across cohorts of patients. (b) Summary of subtype switching between pre and on-treatment samples with PFS time.

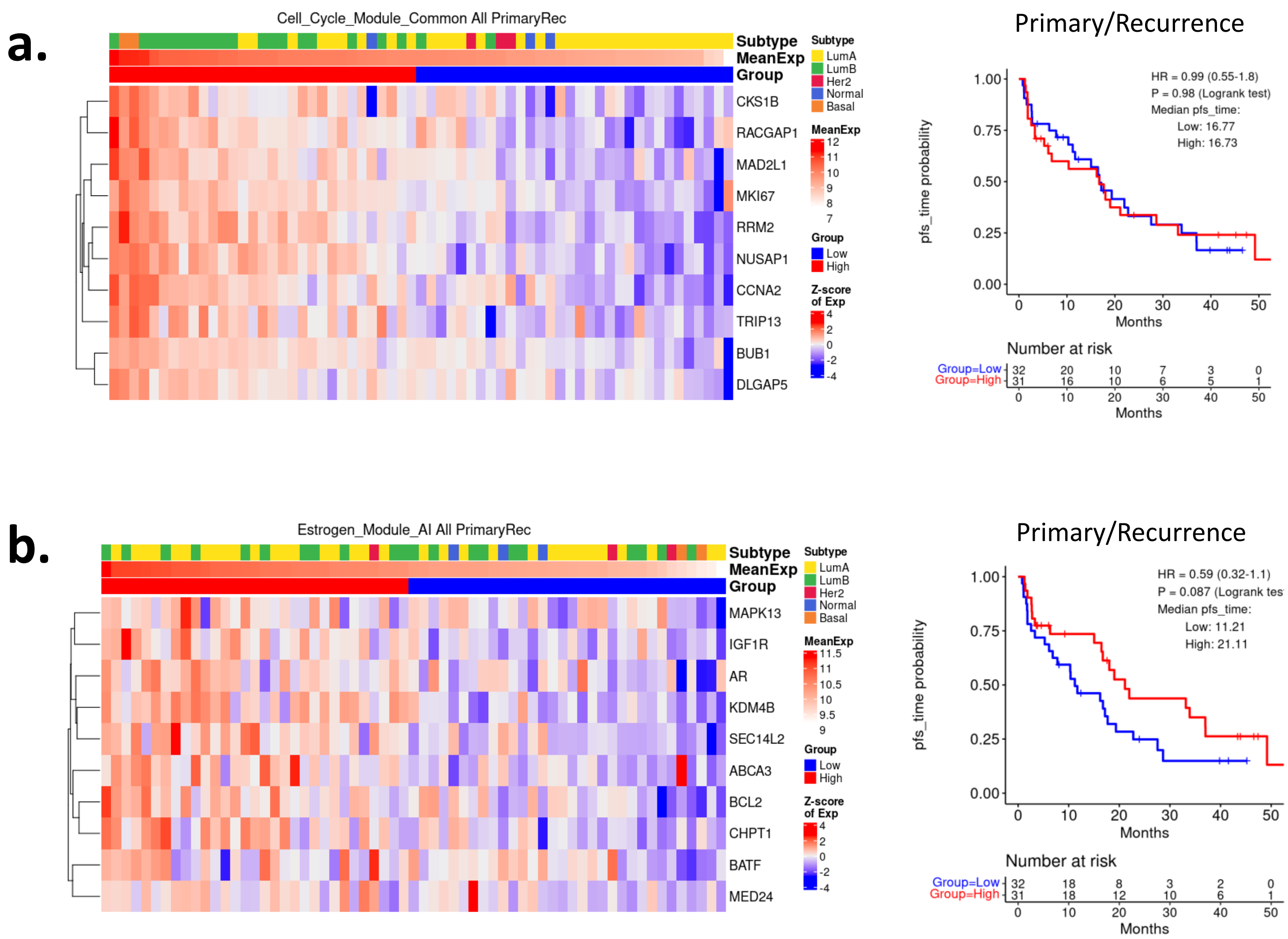


**Supplementary Figure 9.** (a) Heatmap showing gene expression of common cell cycle module in combined AI and Fulvestrant primary/recurrence biopsies and K-M plot comparing PFS between the high and low average gene expression groups using data from primary/recurrent biopsies. p=0.98 by log-rank. (b) Heatmap showing gene expression pattern of the estrogen response module in combined AI and Fulvestrant primary/recurrence biopsies and the associated K-M plot. p=0.087 by log-rank.

Supplementary Figure 10

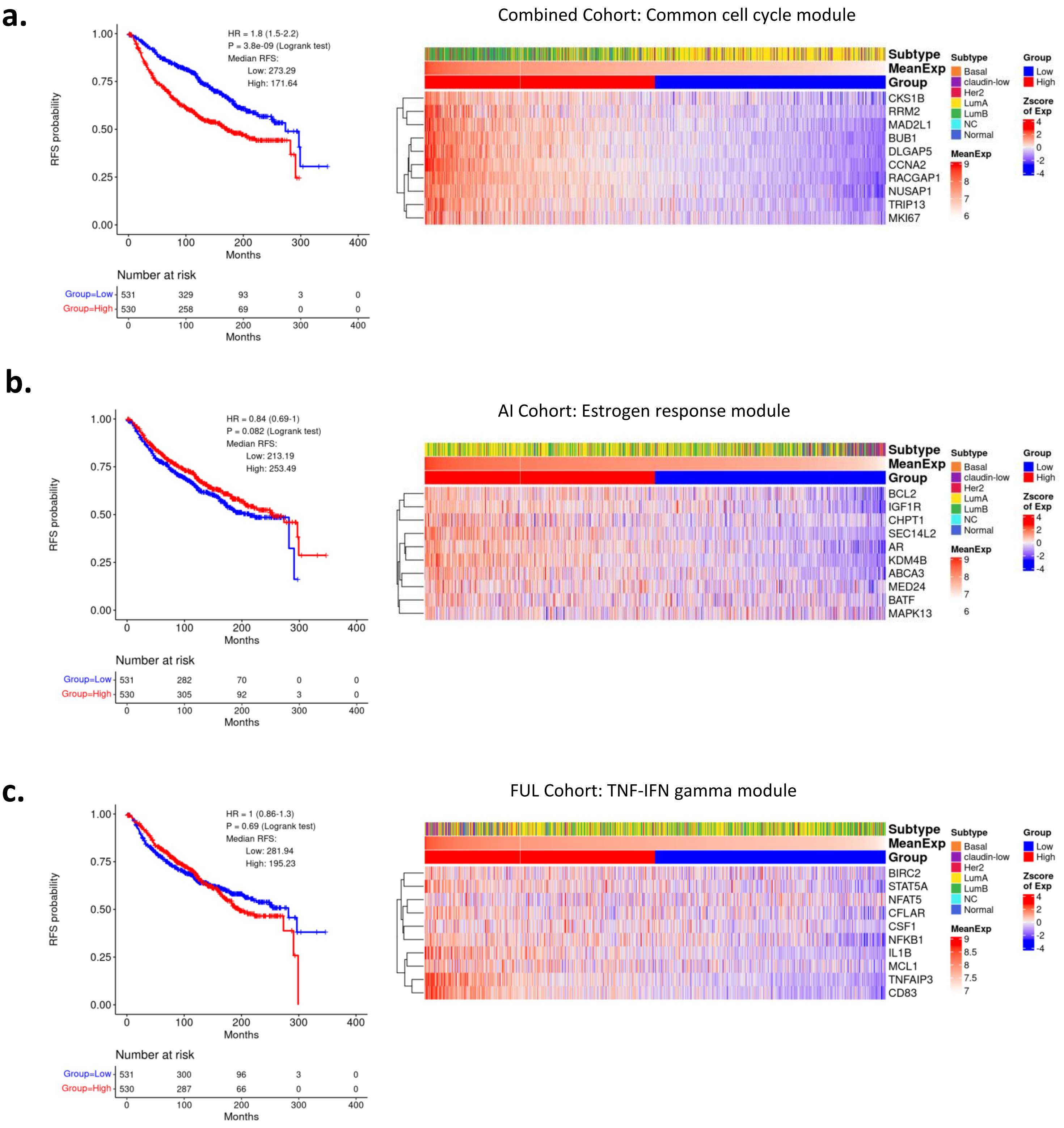


**Supplementary Figure 10.** Evaluation of gene modules on recurrence-free survival on a subset (ER+/HER2-) of the METABRIC dataset. Patients are stratified by mean expression values of the genes in each module. (a) Common cell cycle module. p<0.001 by log-rank. (b) Estrogen response module. p=0.082 by log-rank. (c) TNF-IFN gamma module. p=0.69 by log-rank.

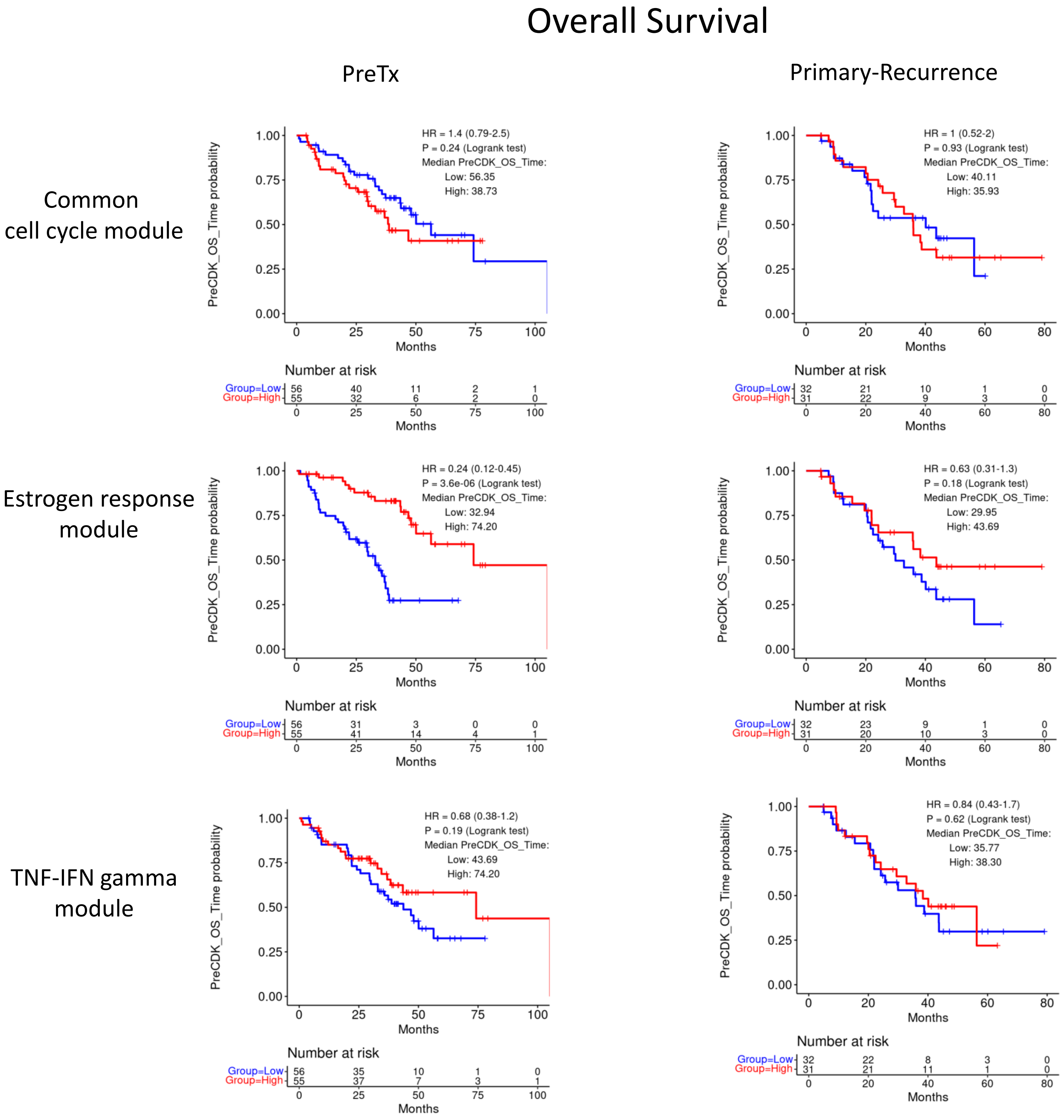


**Supplementary Figure 11.** Evaluation of gene modules on patient overall survival using gene expression data from pre-treatment or primary/recurrence samples. For common cell cycle, p=0.24 and p=0.93 by log-rank, respectively. For estrogen response, p<0.001 and p=0.18 by log-rank, respectively. For TNF-IFN gamma, p=0.19 and p=0.62 by log-rank, respectively.
