# Supplementary material for: Determinants of response to CDK4/6 inhibitors in the real-world setting
Source: NPJ Precis Oncol. 2023 Sep 13;7:90. doi: 10.1038/s41698-023-00438-0 (PMC10499925; doi:10.1038/s41698-023-00438-0)
Supplement: Supplementary file 2 — Reporting Summary [file 41698_2023_438_MOESM2_ESM.pdf]

nature portfolio

Corresponding author(s): Erik Knudsen

Last updated by author(s): Aug 3, 2023

# Reporting Summary

Nature Portfolio wishes to improve the reproducibility of the work that we publish. This form provides structure for consistency and transparency in reporting. For further information on Nature Portfolio policies, see our Editorial Policies and the Editorial Policy Checklist.

## Statistics

For all statistical analyses, confirm that the following items are present in the figure legend, table legend, main text, or Methods section.

| n/a | Confirmed | |
|---|---|---|
| ☐ | ☒ | The exact sample size (*n*) for each experimental group/condition, given as a discrete number and unit of measurement |
| ☐ | ☒ | A statement on whether measurements were taken from distinct samples or whether the same sample was measured repeatedly |
| ☐ | ☒ | The statistical test(s) used AND whether they are one- or two-sided<br>*Only common tests should be described solely by name; describe more complex techniques in the Methods section.* |
| ☐ | ☒ | A description of all covariates tested |
| ☒ | ☐ | A description of any assumptions or corrections, such as tests of normality and adjustment for multiple comparisons |
| ☐ | ☒ | A full description of the statistical parameters including central tendency (e.g. means) or other basic estimates (e.g. regression coefficient) AND variation (e.g. standard deviation) or associated estimates of uncertainty (e.g. confidence intervals) |
| ☐ | ☒ | For null hypothesis testing, the test statistic (e.g. *F*, *t*, *r*) with confidence intervals, effect sizes, degrees of freedom and *P* value noted<br>*Give P values as exact values whenever suitable.* |
| ☒ | ☐ | For Bayesian analysis, information on the choice of priors and Markov chain Monte Carlo settings |
| ☒ | ☐ | For hierarchical and complex designs, identification of the appropriate level for tests and full reporting of outcomes |
| ☒ | ☐ | Estimates of effect sizes (e.g. Cohen's *d*, Pearson's *r*), indicating how they were calculated |

*Our web collection on statistics for biologists contains articles on many of the points above.*

## Software and code

Policy information about availability of computer code

| | |
|---|---|
| Data collection | NA |
| Data analysis | All of the software utilized is open-source and described in detail in the Methods Section. |

For manuscripts utilizing custom algorithms or software that are central to the research but not yet described in published literature, software must be made available to editors and reviewers. We strongly encourage code deposition in a community repository (e.g. GitHub). See the Nature Portfolio guidelines for submitting code & software for further information.

## Data

Policy information about availability of data

All manuscripts must include a data availability statement. This statement should provide the following information, where applicable:

- Accession codes, unique identifiers, or web links for publicly available datasets
- A description of any restrictions on data availability
- For clinical datasets or third party data, please ensure that the statement adheres to our policy

*Provide your data availability statement here.*

## Research involving human participants, their data, or biological material

Policy information about studies with human participants or human data. See also policy information about sex, gender (identity/presentation), and sexual orientation and race, ethnicity and racism.

| | |
|---|---|
| Reporting on sex and gender | Participants in the study include men and women. |
| Reporting on race, ethnicity, or other socially relevant groupings | Not applicable |
| Population characteristics | The data are provided in Figure 1 |
| Recruitment | The participants were prospectively consented to participate in the clinical study<br>An IRB approved tissue acquisition protocol was also employed retrospectively |
| Ethics oversight | IRB at Roswell Park Cancer Center |

Note that full information on the approval of the study protocol must also be provided in the manuscript.

# Field-specific reporting

Please select the one below that is the best fit for your research. If you are not sure, read the appropriate sections before making your selection.

☒ Life sciences ☐ Behavioural & social sciences ☐ Ecological, evolutionary & environmental sciences

For a reference copy of the document with all sections, see nature.com/documents/nr-reporting-summary-flat.pdf

# Life sciences study design

All studies must disclose on these points even when the disclosure is negative.

| | |
|---|---|
| Sample size | The sample size was not pre-determined |
| Data exclusions | Data was excluded when the quality control for gene expression failed, otherwise all data was included. |
| Replication | The clinical samples do not provide the ability for replication. |
| Randomization | There is no randomization in the study. Pairwise assessment was performed for the paired samples we had available. |
| Blinding | No blinding. |

# Reporting for specific materials, systems and methods

We require information from authors about some types of materials, experimental systems and methods used in many studies. Here, indicate whether each material, system or method listed is relevant to your study. If you are not sure if a list item applies to your research, read the appropriate section before selecting a response.

### Materials & experimental systems

| n/a | Involved in the study | |
|---|---|---|
| ☐ | ☒ | Antibodies |
| ☒ | ☐ | Eukaryotic cell lines |
| ☒ | ☐ | Palaeontology and archaeology |
| ☒ | ☐ | Animals and other organisms |
| ☐ | ☒ | Clinical data |
| ☒ | ☐ | Dual use research of concern |
| ☒ | ☐ | Plants |

### Methods

| n/a | Involved in the study | |
|---|---|---|
| ☒ | ☐ | ChIP-seq |
| ☒ | ☐ | Flow cytometry |
| ☒ | ☐ | MRI-based neuroimaging |

## Antibodies

| | |
|---|---|
| Antibodies used | a. MCM2, BioSb, BSB6334, RBT-MCM2, 6336QLFD8<br>b. pRB, Cell Signaling, 8516, Ser807/811(D20B12), lot 9<br>c. RB, Cell Signaling, 9309, 4H1, lot 14 |

d. CCNA2, Abcam, ab32386, X193, GR3208780-5
e. CCND1, ThermoFisher,MA1-39546, SP4, XJ3739161
f. CCNE1, Abcam, ab33911, EP435E, GR3324969-1
g. Cytokeratin, Dako, M3515, AE1/AE3, 11195026
h. Ki67, Abcam, ab16667, SP6, GR3375640-35

Validation

a. We validated all primary antibodies for human tissue using appropriate positive and negative controls. We first stained each primary antibody using traditional DAB immunohistochemistry. We titrated primary antibody dilutions using manufacturer recommended conditions and the Human Protein Atlas as our references. We than had a pathologist confirm the staining. We then moved the optimization to multispectral, pairing each antibody with a opal fluor and repeating the process until IF values for each marker were in the optimal range of 10:1 as compared to background. Again, the final panel staining results were verified by a pathologist. Please reference our methods section in the paper for further detail.

## Clinical data

Policy information about clinical studies

All manuscripts should comply with the ICMJE guidelines for publication of clinical research and a completed CONSORT checklist must be included with all submissions.

Clinical trial registration

NCT04526587

Study protocol

ClinicalTrials.gov

Data collection

All patient data was collected from a review of patient charts at Roswell Park Comprehensive Cancer Center between July 2020 and December 2022.

Outcomes

PFS and OS were based on data from electronic medical record.
